# Design Engineering, Synthesis Protocols, and Energy Applications of MOF-Derived Electrocatalysts

**DOI:** 10.1007/s40820-021-00656-w

**Published:** 2021-06-01

**Authors:** Amr Radwan, Huihui Jin, Daping He, Shichun Mu

**Affiliations:** 1 School of Science, Wuhan University of Technology, Wuhan, 430070 People’s Republic of China; 2 Foshan Xianhu Laboratory of the Advanced Energy Science and Technology Guangdong Laboratory, Xianhu Hydrogen Valley, Foshan, 528200 People’s Republic of China; 3 State Key Laboratory of Advanced Technology for Materials Synthesis and Processing, Wuhan University of Technology, Wuhan, 430070 People’s Republic of China

**Keywords:** MOF-derived electrocatalysis, Oxygen reduction reaction, Oxygen evolution reaction, Hydrogen evolution reaction

## Abstract

The core reactions for fuel cells, rechargeable metal–air batteries, and hydrogen fuel production are the oxygen reduction reaction (ORR), oxygen evolution reaction (OER), and hydrogen evolution reaction (HER), which are heavily dependent on the efficiency of electrocatalysts. Enormous attempts have previously been devoted in non-noble electrocatalysts born out of metal–organic frameworks (MOFs) for ORR, OER, and HER applications, due to the following advantageous reasons: (i) The significant porosity eases the electrolyte diffusion; (ii) the supreme catalyst–electrolyte contact area enhances the diffusion efficiency; and (iii) the electronic conductivity can be extensively increased owing to the unique construction block subunits for MOFs-derived electrocatalysis. Herein, the recent progress of MOFs-derived electrocatalysts including synthesis protocols, design engineering, DFT calculations roles, and energy applications is discussed and reviewed. It can be concluded that the elevated ORR, OER, and HER performances are attributed to an advantageously well-designed high-porosity structure, significant surface area, and plentiful active centers. Furthermore, the perspectives of MOF-derived electrocatalysts for the ORR, OER, and HER are presented.

## Highlights

- Synthesis protocols, design engineering, theoretical calculations, and energy applications for metal–organic frameworks (MOFs)-derived electrocatalysts are systematically analyzed.
- Synthesizing methods of MOF-derived catalysts and their oxygen reduction reaction, oxygen evolution reaction, and hydrogen evolution reaction electrocatalysis are discussed.
- The current status, ongoing challenges, and potential future outlooks of MOFs-derived electrocatalysts are highlighted.

## Graphical abstract

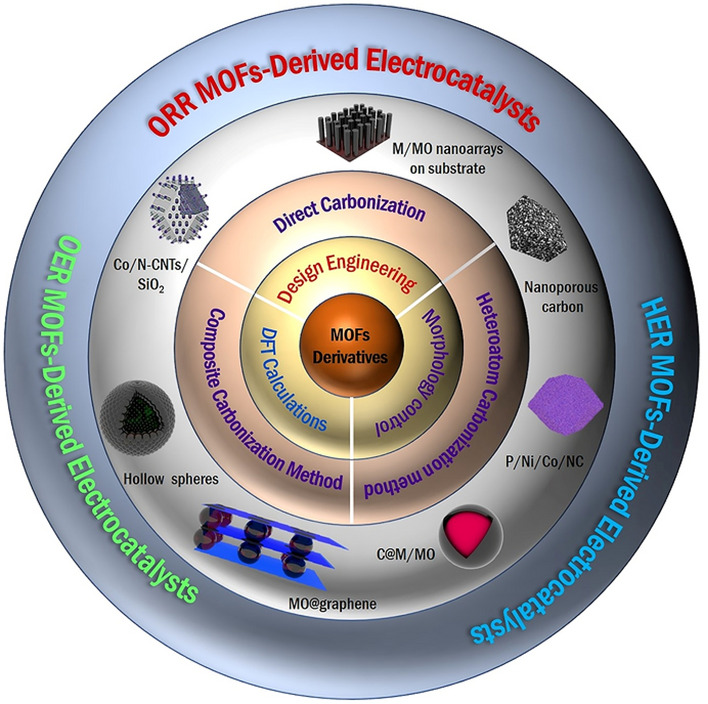

## Introduction

Currently, global warming and air pollution have become two critical worldwide tasks in the twenty-first-century civilization caused by fossil fuel during production and use, which can be resolved only by using alternative environmentally friendly energy sources. Over the last decades, increasing endeavors have been devoted to energy alternative sources in particular wind energy [1, 2], hydroelectric power [3–8], hydrogen energy [9–11], solar energy [12], and nuclear energy [13], which have already been served as complementary sources of energy to the traditional fossil fuels. One of the most promising is eco-friendly hydrogen energy and its applications for hydrogen fuel production, and hydrogen fuel cells [14–16]. For instance, hydrogen fuel can be generated by water splitting and utilized without the generation of harmful NO_x_ or CO_2_; meanwhile, hydrogen has a significantly higher energy density of 120 MJ kg^−1^ comparative with the gasoline of 44 MJ kg^−1^. Besides, hydrogen fuel cells exhibit energy density up to four times higher than that of batteries. Also, metal–air batteries have been developed as highly efficient energy application systems [17–21], owing to the high Li–air battery theoretical energy (11.140 Wh kg^−1^) relative to gasoline (12.200 Wh kg^−1^) [22–25]. However, the aforementioned energy application systems, embracing the oxygen reduction reaction (ORR), oxygen evolution reaction (OER), or hydrogen evolution reaction (HER), are vigorously dependent on the appropriate type of catalysts.

**Fig. 1:**
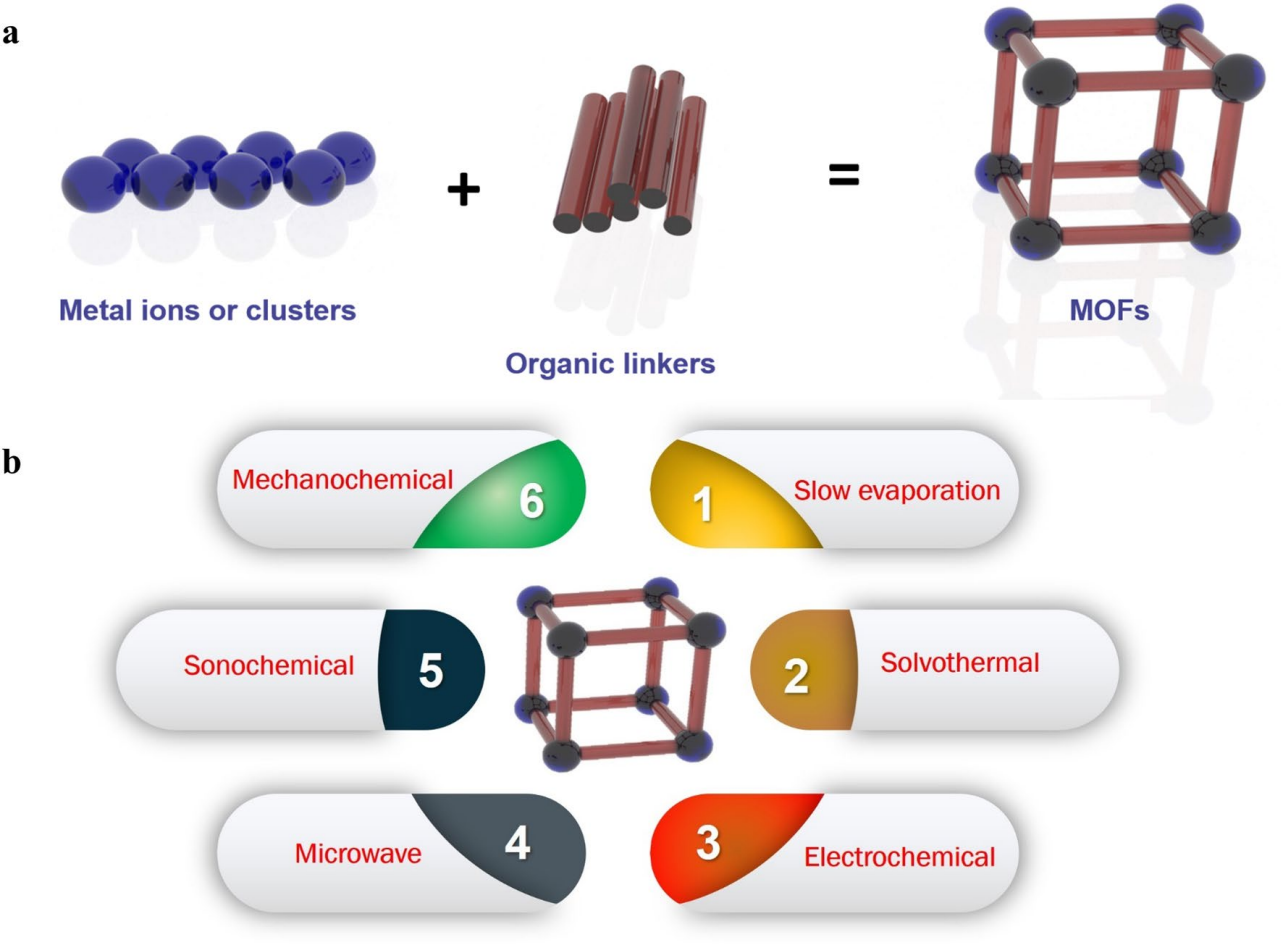
**a** Illustration of the building blocks and structure of MOFs. **b** Methods commonly used for as-prepared MOF synthesis

The oxygen reduction is classified among the essential half-reactions that occur in several energy applications such as metal–air batteries and fuel cells [14, 15, 17, 18, 20, 26–44] (Fig. 1). Nevertheless, the slow kinetics of ORR, high-priced (Pt or Pd), and the inadequate stability of catalysts are the most crucial limitations to such energy conversion applications [45, 46]. Thus, searching for electrocatalysts with superior efficiency for ORR is a significant milestone to broaden the marketing of these progressive energy storage systems. As part of the review, numerous significant researches have been carried out for Pt-free-based catalysts for ORR in fuel cells like emerging extremely active non-noble metal or cheap metal oxide electrocatalysts, and exploring novel supporting materials to enrich the active site centers. Among these advantageous electrocatalysts, high-porosity carbons were extensively used in numerous fields of electrode materials for energy conversion and storage systems. The attractive characteristics for porous carbon-based materials are high conductivity, exceptional surface area, abundant porous nature, cost-effectiveness, and outstanding anti-corrosion properties, which can be taken into account to be the best ideal supports for Pt-free catalysts for ORR in fuel cells. To get porous carbon-based electrocatalysts with extraordinary performance, numerous methods, such as direct pyrolysis for carbonaceous precursors (such as polymeric aerogels and organic materials), have been tried. Meanwhile, the disordered activated carbon might have low availability thanks to the pore size distribution. Consequently, it is crucial to discover a suitable platform material for carbon-based electrocatalysts having extraordinary specific surface areas, well-ordered morphology, large formed pores, and appropriate ORR chemical stabilities.

The oxygen evolution is a crucial half-reaction for the water electrolysis (water splitting) and metal–air batteries. Precious metals, particularly Ir and Ru, and their oxides have been widely applied as highly active electrocatalysts for OER; meanwhile, their slow kinetics, significant cost, and insufficiency certainly forbid their widespread application [47, 48]. Consequently, great endeavors have been dedicated to applying earth-abundant metals-derived electrocatalysts, for instance, metal phosphates (PO_4_)^2−^, hydroxides (OH)^−^, oxides (O)^2−^, chalcogenides (S, Se, Te), and nitrides (N)^3−^, to promote the OER performance [49–52]. Nevertheless, their catalytic activity is still restricted by their low conductivity and inadequate active surface area, leading to limited kinetics and high OER overpotentials. Therefore, most studies aspire to expand the active site numbers exposed to the electrolyte and raising the intrinsic efficiency. Numerous approaches have been investigated and applied. Nevertheless, high loading and open framework are vigorous to rise the exposed active site numbers, whereas the compositions with controlled design through simple itineraries are predictable to strengthen the practical activity. It is still demanding to instantaneously increase the active site numbers and rise the inherent activity to implement an extremely effective oxygen electrocatalyst.

The hydrogen evolution is another vital half-reaction that occurs throughout the water electrolysis [53]. Presently, Pt is still the most applied HER catalyst with very low overpotentials. Nevertheless, the limited availability and the high-priced Pt restrict its implementation in water electrolysis large scale. Thus, emerging highly active precious-metal-free catalysts for HER has a vital significance issue to assist the spread of electrochemical hydrogen implementations.

**Fig. 2:**
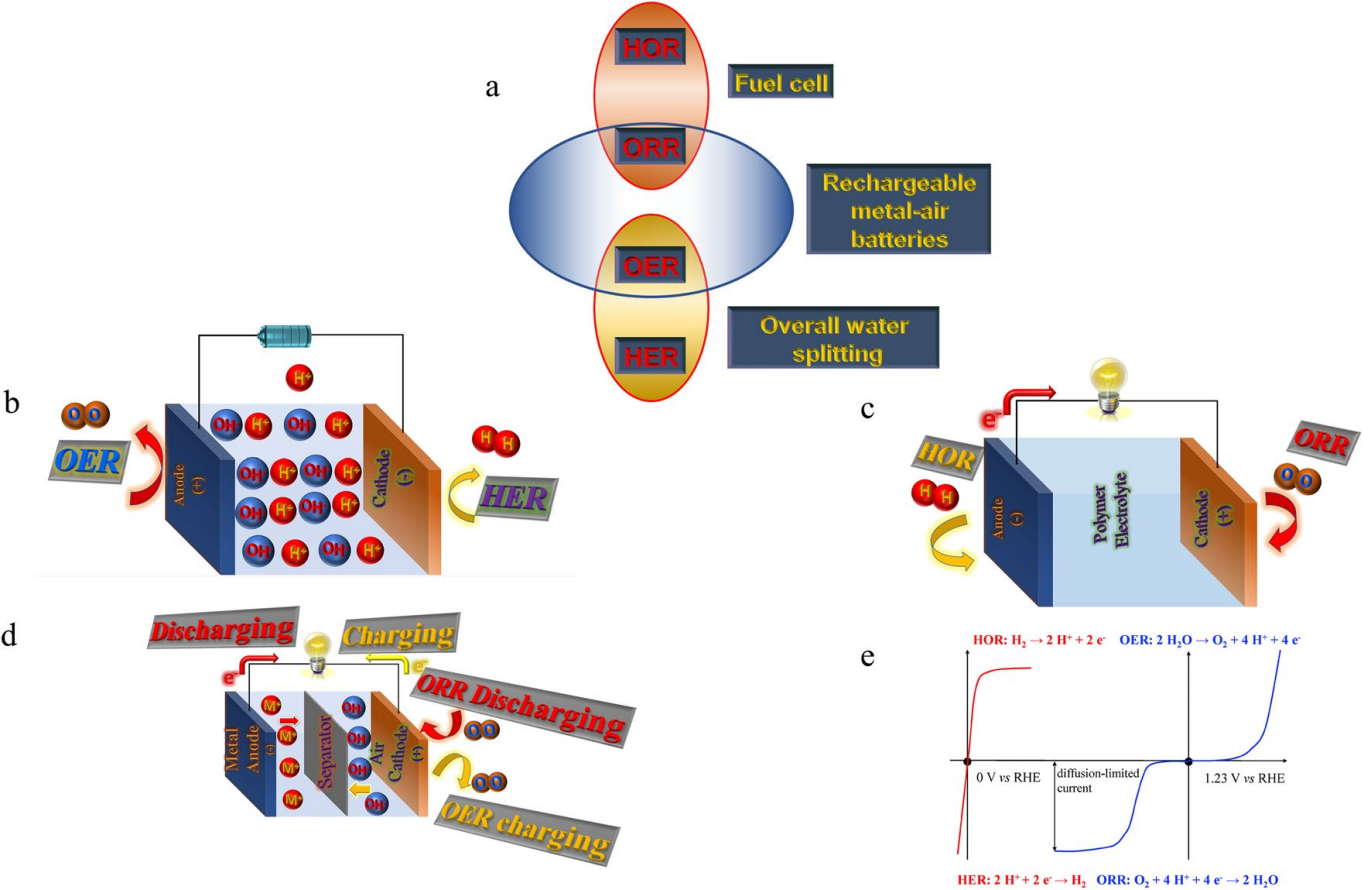
Schematic illustration of: **a** reactions in fuel cell, metal–air batteries and water electrolysis, **b** water electrolyser, **c** fuel cell, **d** metal–air batteries. **e** Current–potential characteristics of ORR, OER, HOR, and HER [167], Copyright © 2015, The Royal Society of Chemistry

Metal–organic frameworks (MOFs), with ideal forms and suitable functional groups able to facilitate the reactions through the substance transport in the energy storage or conversion progressions, are promising unique platforms for electrocatalysis with extremely ordered carbon-based materials and high porosity [54, 55]. A set of high porosity with inherent nanopores (commonly pore with sizes less than 100 nm) offer an auspicious path to develop and prepare highly well-organized oxygen electrocatalysts. Also, MOFs consist of inorganic subunits such as layers, clusters, chains, or 3D arranging coupled to organic linkers (spacers) with complexing groups (phosphonates, carboxylates, N-comprising compounds) via robust bonds (Fig. 2). This outcomes in 3D mixed frameworks where ingredients from both organic and inorganic become available. Practically, a lot of elements in the periodic table can be linked to the creation of single or numerous MOF frameworks. Significant numbers of organic spacers can be utilized to incorporate several organic linkers, aromatics, or aliphatics, occasionally replaced by heteroatoms (nitrogen, oxygen, sulfur, etc.), or connected with one or more complexing functions that might be anionic or neutral (carboxylates, sulphonates, phosphonates, imidazolates, amides, amines, pyridyls, nitriles groups, or a combination of them). Thanks to the periodic table richness with the metal element chemistry and organic chemistry, MOFs investigated in the literature are presently expanding consistently. Consequently, usually every short period, novel researches for synthetic MOFs containing various pore sizes, novel shapes, and organic functionalities become available. Inside the 3D structure, the MOF cavities or channels are filled with the molecules of solvent or spacer-free molecules that could quickly be lost by thermal treatments, with/without vacuum. MOFs have key advantages owing to their ultrahigh porosity up to 90% free volume and massive inner and outer surface, extending to 6000 m^2^ g^−1^ [56]. MOFs are typically produced by solution mixture from room temperature to 250 °C at ambient pressure, with reaction time from a couple of minutes to several days. Due to the presence of the organic and inorganic moieties in their frameworks, they were extensively investigated in several technological applications such as gas adsorption and separation [57–59], electrocatalysis [17–19, 60, 61], chemical sensing [62–64], biomedical applications [64, 65], and proton conduction [66, 67]. Importantly, owing to the presence of the organic and inorganic counterparts in their composition, MOFs present numerous application chances in catalysis. Impressively, in comparison with modified mesoporous silica or zeolites, MOFs have the ability for the direct combination of catalytic active metals and the simple change of the neighborhood of the catalytic active centers across the functionalized linkers even though their relatively low stability stays a significant disadvantage [68–70]. To implement an extraordinary catalytic activity, two catalyst design protocols have been established by increasing the reachable active site numbers of catalyst materials and rising the inherent performance of each active site which could be controlled in MOFs. Undoubtedly, for ORR, OER, and HER, MOFs could afford a suitable solution for non-precious electrocatalysis implementations. In consonance with these guidelines, various MOF-derived materials have been reported in the past few years. Nevertheless, a comprehensive review summarizing the recent MOF-derived electrocatalysis with well-defined synthetic protocols, design engineering, reaction mechanisms, morphologies, electrocatalytic activities, and DFT calculation analysis is urgently needed to provide strong inspiration and direct future expansions in engineering for ORR/OER/HER MOF-derived electrocatalysts.

Herein, this review is designed for summarizing the current progression in the design and engineering structure of MOF-derived catalysts for energy conversion applications. Significantly, through this detailed review, we highlight the novel design, highly active, and durable carbon-derived electrocatalyst synthesis, depending on the superiority of MOFs precursors in the interior of the framework, and also demonstrate the electrochemical efficiency for ORR, OER, and HER. We believe that this review will be a valuable reference for researchers who are focusing on the fields of MOF-derived electrocatalysis and others. Moreover, this review will stimulate extensive interest to further accelerate and discover the innovations of MOF-derived materials in ORR/OER/HER-related energy technologies.

## Design Engineering of MOF-Originated Materials

### Design Engineering

**Fig. 3:**
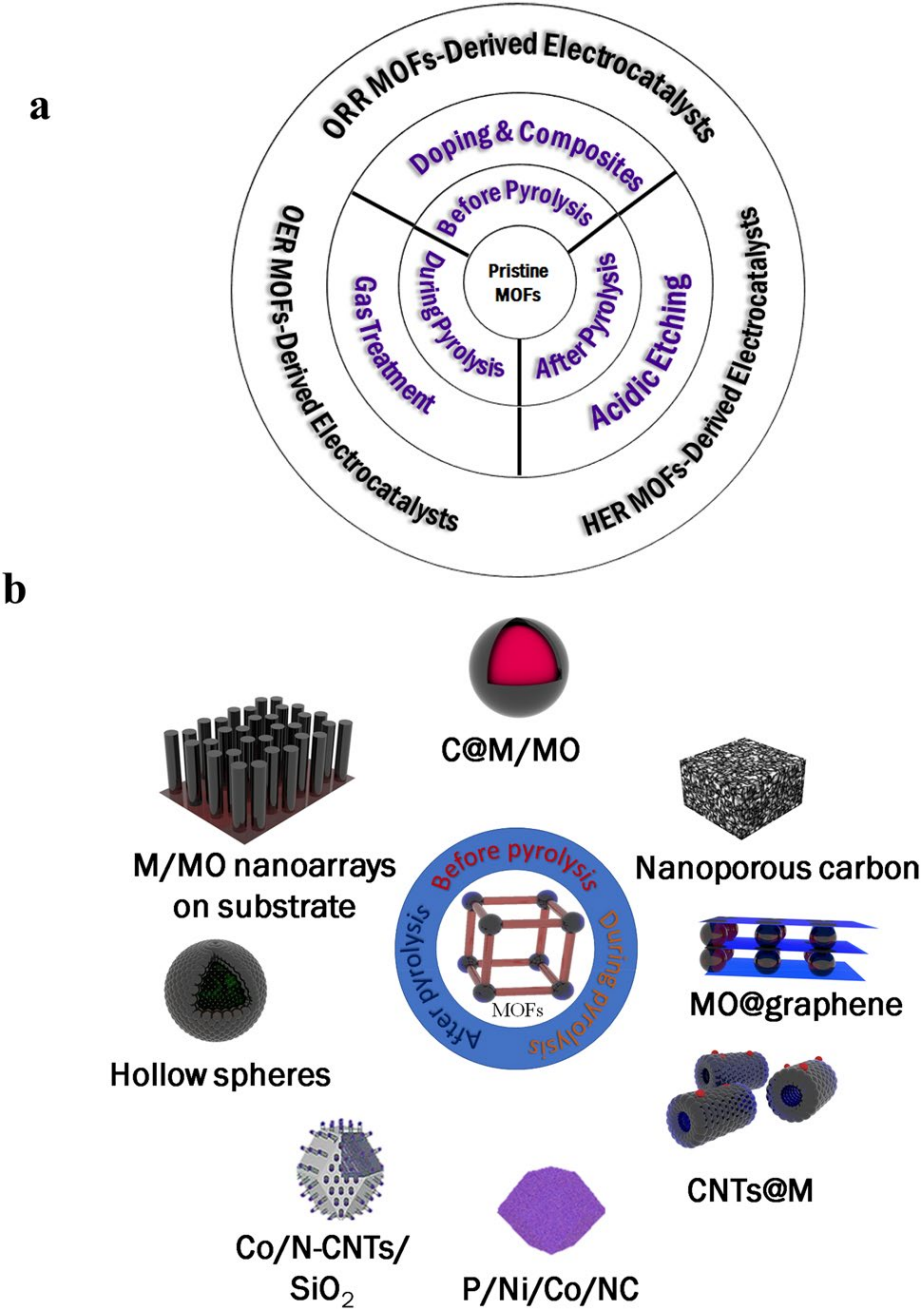
**a** A schematic summary for general synthesis protocols for advanced electrocatalysts derived from MOFs. **b** Descriptive scheme of tailoring MOFs and MOF-based materials

In the beginning, it is important to identify MOF-derived materials to focus on the context of the discussion. In this scope, MOF-derived materials will be particular for platforms obtained from MOF precursors through a set of post-treatment as thermal treatment, chemical modification, and surface decorating (Fig. 3). Briefly, their synthesis occurs by selective preparation of a MOF precursor followed by the pyrolysis treatment for this precursor. Generally, MOFs are nearly entirely obtained by hydrothermal/solvothermal or by traditional solution-based approaches. Also, the MOF precursor fabrication has been explained in detail in several types of research [71, 72]. Herein, our focus will be mainly on the MOF precursor treatment utilized to acquire aimed materials with desired structures and morphologies (like core–shell structures, hollow nanowire array structures, sandwich structures, and 3D hierarchical structures). Because the post-processing is to achieve a catalyst with definite features that enhance electrochemical performance, it becomes vital to illuminate the common correlation among the catalytic activity material and physicochemical properties. Firstly, the inherent characteristics of material control its efficiency and conductivity. Sensible choice of constituents is advantageous in decreasing overpotentials, decreasing Tafel slopes, and rising catalytic current density. Meanwhile, the integration of constituents with high conductivity eases quick electron transport. Secondly, the modified morphology of the material can exhibit an increase in the active site numbers, particularly definite crystal faces with high activity. Lastly, the construct is tightly linked with the active center numbers and robustness as favorable compositions influence in preventing accumulation and boosting mass transfer during reactant diffusion and product propagation. In light of this, the designed materials of MOF-derived catalysis will emphasize these three features [71].

Structural modulation is very efficient in electrocatalysis applications due to offering increasing in the exposed active site number because of the rising in the surface area. But, intrinsic catalysts without carbon protection can be corroded by the strong base or acid electrolytes. As a consequence, the core–shell structure could be a suitable solution for structural engineering. This is because, for instance, the H adsorption free energy is extremely high on the cobalt atoms and extremely weaker on the carbon and then it can show a more ideal outcome on the core–shell as a consequence of the electronic movement through cobalt core to carbon shell [73]. In addition, a MOF-derived reverse-encapsulation structure where the unstable phase or composite is protected by a stable phase as an outer layer, such as Co-NC@Mo_2_C complex, is beneficial for efficient electrocatalysis [60].

### Structure and Morphology Control Challenges

Besides the design of structures and morphologies, how to control them is still an additional critical key in constructing high-efficiency catalysts derived from MOFs, as it directly links with exposed facets and active sites. The activity of each facet differs extensively because of their diverse electronic structures, as it is shown through the ORR catalytic performance for the Pt low index surfaces diminishes by the following direction: (110) > (111) > (100) [74, 75]. Therefore, controlling the objective morphological structure is a significant approach for rising the inherent activity throughout exposing active facets in addition to sites. Nevertheless, the morphology and structure of the precursor MOFs become destroyed after thermal treatments at elevated temperatures, thanks to acute and permanent shrinking, fusion, pulverization, and accumulation. These alterations can negatively influence catalytic activity. It is very well known that aggregation often causes a significant reduction in the active site numbers with poor dispersibility, reducing activity in addition to rapid mass transfer. Likewise, the size reduction and fusion of MOFs usually result in the deterioration of their porous framework and subsequently hinder the fast accessibility from reactants to active centers and the diffusion of reaction outputs. Taking into consideration the aforementioned above, it can be concluded that the morphology and structure control exhibits a considerable strategy to design highly effective catalysts acquired from MOFs. Nevertheless, it becomes a big task to protect the MOFs precursors throughout the pyrolysis processes.

Presenting an outer shield as a defensive covering has been utilized in nanotechnology to stop or decrease the aggregation, particularly when high-temperature calcination is included, as it was first reported by our group’s calcination protection approach throughout coating with mesoporous silica (mSiO_2_) [19]. Through this study, Co-based MOFs (ZIF-67), with a well-defined dodecahedral shape in addition to a soft surface with a particle size of ∼ 600 nm, have been utilized to test the method. As shown in Fig. 5a, through the existence of mSiO_2_ film externally, the ZIF-67 crystals can stop the Co catalysts from the rapid accumulation and extensively contributes like an exceptional ‘strainer’ for reminding CNTs catalytic growth throughout the thermal treatment. The better-covered forms with their ideal intrinsic qualities permit an enlarged number of active centers, in addition to that facilitate mass transfer processes, by this means giving extraordinary catalytic efficiency and outstanding stability. Moreover, to acquire a form of concave Fe–N–C single-atom catalysts having an improved mesoporosity and exterior surface area, the anisotropic thermal reduction of ZIF-8 was implemented to obtain the concave morphology [76]. Concerning the outside SiO_2_ covering layer, the dodecahedron’s edge frame becomes preserved during the planar faces collapsed. Afterward, thermal treatment for SiO_2_-coated ZIF-8 nanoparticles at 650 °C followed by etching for the SiO_2_ shell, which produced an extremely defective host material having concave structures with enlarged micropores. Nevertheless, the complex synthesis process and the employment of corrosive with toxic HF throughout the SiO_2_ shell attacking make that problematic for actual implementation. Consequently, emerging simple and real approaches for controlling structure is further an immense task that desires to be tackled.

## Synthesis Protocols for MOF-Derived ORR, OER, and HER Catalysts

The major synthesis protocols and the obtained MOFs-derived electrocatalysts for ORR, OER, and HER are depicted in Fig. 3a. For synthesis protocols, it always uses MOFs as sacrificial templates; after pyrolysis, numerous classes of derivatives involving carbon nanostructure [77], metal oxides [78], metal composites (M/MO@C) [79], metal carbides (MC) [80], and metal nitrides (MN) can be offered. MOFs-derived electrocatalysis can be functionalized during, before, or after pyrolysis. Additionally, the heteroatom doping with nitrogen (N), phosphorous (P), boron (B), etc., becomes recognized to enhance the catalytic response in an exponential manner in carbon-based metal-free and non-precious M/C hybrid electrocatalysts. Such MOF derivatives significantly outspread the implementation of MOF-based catalysts, keeping off some critical disadvantages of direct-MOF electrocatalysts. Overall, the as-prepared MOFs show low robustness in extremely acidic or alkaline aqueous-based electrolytes, which are conventional in electrochemical systems, whereas derivatives from MOFs display far more robustness under severe working environments [81–84]. Furthermore, elucidative synthesis, design engineering, and energy applications of materials derived from MOFs will be presented in the subsequent sections.

Through ORR catalysts, three major categories will be investigated: (i) nonmetal heteroatom-doped porous carbon catalysts derived from MOFs materials; (ii) monometallic-MOF derivative-nanocarbon electrocatalysts, and (iii) bimetallic/multimetallic-MOFs-based electrocatalysts. Nitrogen-doped carbon was noticed to be the extremely generally explored nanomaterials for the ORR in contrast to heteroatoms. Through this section, synthesis protocols for nitrogen-doped carbon will be covered, while the other categories will also be covered through the review. General synthesis protocols for previous studies can be assigned into three kinds: (i) the pyrolysis of MOFs containing nitrogen such as zeolitic imidazolate frameworks ZIFs, which is nominated as *in situ* nitrogen doping; (ii) the amendment approach for carbonization processing after synthesis through utilizing gas guest molecule such as ammonia, which is nominated as *ex situ* nitrogen doping; (iii) using second solid material as a nitrogen source mixed with MOFs, which can produce an *in situ* nitrogen-doped carbon with bulky surface area and controlled pore size distribution. Several organic materials, for example, melamine, furfuryl alcohol (FA), and glucose, are utilized to accomplish function [85–87]. Shortly, the organic material used as a source for both nitrogen and carbon enters inside the holes for the MOFs template at the beginning, followed by the pyrolysis process, producing nanocarbon doped with nitrogen. Herein, the second source of carbon addition such as AF not only extensively enhances the resultant graphitized nanocarbon but also eliminates the metal species; through this fabrication process, metal-free nanoporous structure electrocatalyst could be obtained. Moreover, as an instant precursor, MOFs are utilized to obtain heteroatom-doped porous carbon electrocatalysts thanks to the existence of several heteroatoms in the organic ligand and also rich with carbon. Through direct pyrolysis, MOFs are typically employed as a mold, whereas the nitrogen-doped carbon could be obtained during the structure precursor transformation for the MOFs bearing nitrogen. Consequently, MOFs have been applied as excellent precursors to having carbon-based catalysts owing to the presence of homogenous dispersed catalytic centers and dense active sites.

Furthermore, MOFs-derived materials for OER catalysts contain three categories as follows: (i) metal-free OER electrocatalyst; (ii) MOF-derived metal-involved electrocatalysts; and (iii) MOF-derived metal oxide catalysts, whereas MOFs-derived materials for HER catalysts contain (i) MOF-derived transition metal/heteroatoms (P, S, O, C, Se) and (ii) MOF-derived metal-based electrocatalysts; all these groups and subgroups will be explained in detail through the review. In this section, the three major synthesis methods will be discussed as follows:

### Direct Carbonization Method

Recently, one of the most extensive methods used for obtaining MOF-derived ORR, OER, and HER catalysts is the direct carbonization method [88, 89]. Through this method, numerous MOFs were applied such as Co‐MOF [90], Ni‐MOF [91], and Zn-MOF [77]. According to the method, researchers can have the ability to control the size and morphology of the resultant catalysts, which facilitate the active sites of as-prepared catalysts analysis. Nevertheless, the characteristics of the electrocatalysis samples are overwhelmingly restricted by the atom type and the construction of the MOFs template. To overcome the atom type limitation of a MOF precursor, bimetallic MOF materials have been applied as a successful protocol to obtain efficient catalysts through direct carbonization [17]. Owing to the bimetal doping synergetic effect, the efficiency of the catalyst of bimetallic MOF electrocatalysts is usually much better than that of single-containing MOF-derived ones.

### MOF–Heteroatom Source Mixture Carbonization Method

Concerning preparing highly active electrocatalysts, the MOF–heteroatom source carbonization technique is extensively utilized. Via inserting another heteroatom resource to MOFs as precursors, the doping with exterior heteroatom could be prepared rationally and exhibit a second solution for the atom types limitations in the catalysts. The external heteroatom doping sources consist of nonmetal sources (for instance as N, P, S, and C) [92–94] and/or metals source (Fe, Co, Cu, etc.) [27, 95–97]. The majority of the secondary nonmetal dopants incorporate furfural, dicyandiamide, chitosan, carbon, glucose, sucrose, acetonitrile, glycerol, xylitol, tetrachloride, triarylphosphine, glucose, ethylenediamine, urea, dimethyl sulfoxide, cyanamide, thiourea, melamine, thioacetamide, etc. Combining highly different electronegativity nonmetal atoms inside carbon frameworks can successfully adapt the electronic distribution of the charge density of carbon materials, signifying that electronegativity of the nonmetal atoms induces the positive charge to the adjacent carbon atoms and further endorses the catalytic behavior. Furthermore, traces of metal doping would make the bared active site without aggregation, which proficiently strengthens the catalytic activity.

### MOF-Based Composite Carbonization Method

It is widely recognized that carbon electrocatalysts arising out of MOF precursors at elevated temperatures commonly undergo many difficulties. For instance, the skeleton distortion at temperatures over 700 °C and the porosity reduction occurs for MOFs [98]. Remarkably, the composite obtained from MOF-based precursors, such as MOF/carbon nanotubes, and MOF/graphene, become capable of overwhelming the hindrances to obtaining highly efficient catalysts. For instance, carbon coating on MOFs surfaces or merging the MOF with an appropriate templating agent could competently prohibit the MOF skeleton material from breakdown, which indicates that the porous carbon composite catalysts derived from MOF can preserve highly specific surface area (SSA) and durable porousness, which leads an improved conductivity and graphitization degree of the produced materials. Therefore, this method greatly enhances the catalytic efficiency of the catalysts.

## Theoretical Exploration for Related Electrocatalytic Reaction Mechanism

As shown in Fig. 1e, the OER process is simply a reverse version of the ORR process (Table 1). By the reduction for O_2_ to H_2_O or OH, the ORR pathway could occur; meanwhile, by the H_2_O oxidation to O_2_ occurs through the OER pathway. Through numerous effects such as the electrode surface structure, materials with several facets, different reaction mechanisms could happen [99]. The most admitted OER overall reaction pathways include four discrete electron transfer stages, which are listed in Table 1, where * is the catalyst active site. The DFT computations outcomes expose that the HER process probably includes the Volmer–Heyrovsky or Volmer–Tafel route on a range of transition-metal surfaces, whereby the Volmer step is a fast reaction whereas the Heyrovsky or Tafel reaction is assigned to the rate-determining step [100, 101].

**Table 1:** ORR, OER, and HER overall reactions and the reaction pathways

ORR	
(Acidic solution) ${\mathrm{O}}_{2}+{4\mathrm{H}}^{+}+{4\mathrm{e}}^{-}\to {\mathrm{H}}_{2}\mathrm{O}$ (*E*_0_ = 1.23 V vs. RHE)	${\mathrm{O}}_{2}+{2\mathrm{H}}^{+}+{2\mathrm{e}}^{-}\to {\mathrm{H}}_{2}{\mathrm{O}}_{2}$ ${\mathrm{H}}_{2}{\mathrm{O}}_{2}+{2\mathrm{H}}^{+}+{2\mathrm{e}}^{-}\to {2\mathrm{H}}_{2}\mathrm{O}$
(Alkaline solution) ${\mathrm{O}}_{2}+{2\mathrm{H}}_{2}\mathrm{O}+{4\mathrm{e}}^{-}\to {4\mathrm{OH}}^{-}$	${\mathrm{O}}_{2}+{\mathrm{H}}_{2}\mathrm{O}+{2\mathrm{e}}^{-}\to {\mathrm{OOH}}^{-}+{\mathrm{OH}}^{-}$ ${\mathrm{OOH}}^{-}+{\mathrm{H}}_{2}\mathrm{O}+{2\mathrm{e}}^{-}\to {3\mathrm{OH}}^{-}$

OER	
(Acidic solution) ${2\mathrm{H}}_{2}\mathrm{O}\to {\mathrm{O}}_{2}+{4\mathrm{H}}^{+}+{4\mathrm{e}}^{-}$ (*E*_0_ = 1.23 V vs. RHE)	$\star +{\mathrm{H}}_{2}\mathrm{O}\to {\mathrm{OH}}^{\star }+{\mathrm{H}}^{+}+{\mathrm{e}}^{-}$ ${\mathrm{OH}}^{\star }\to {\mathrm{O}}^{\star }+{\mathrm{H}}^{+}+{\mathrm{e}}^{-}$ ${\mathrm{O}}^{\star }+{\mathrm{H}}_{2}\mathrm{O}\to {\mathrm{OOH}}^{\star }+{\mathrm{H}}^{+}+{\mathrm{e}}^{-}$ ${\mathrm{OOH}}^{\star }\to {\mathrm{O}}_{2}^{\star }+{\mathrm{H}}^{+}+{\mathrm{e}}^{-}$ ${\mathrm{O}}_{2}^{\star }\to \star +{\mathrm{O}}_{2}$
(Alkaline solution) ${4\mathrm{OH}}^{-}\to {\mathrm{O}}_{2}+{2\mathrm{H}}_{2}\mathrm{O}+{4\mathrm{e}}^{-}$	$\star +{\mathrm{OH}}^{-}\to {\mathrm{OH}}^{\star }+{\mathrm{e}}^{-}$ ${\mathrm{OH}}^{\star }+{\mathrm{OH}}^{-}\to {\mathrm{H}}_{2}\mathrm{O}+{\mathrm{O}}^{\star }+{\mathrm{e}}^{-}$ ${\mathrm{O}}^{\star }+{\mathrm{OH}}^{-}\to {\mathrm{OOH}}^{\star }+{\mathrm{e}}^{-}$ ${\mathrm{OOH}}^{\star }+{\mathrm{OH}}^{-}\to {\mathrm{O}}_{2}^{\star }+{\mathrm{e}}^{-}$ ${\mathrm{O}}_{2}^{\star }\to \star +{\mathrm{O}}_{2}$

HER	
(Acidic solution) ${2\mathrm{H}}^{+}+2{\mathrm{e}}^{-}\to {\mathrm{H}}_{2}$	Volmer: $\star +{\mathrm{H}}^{+}+{\mathrm{e}}^{-}\to {\mathrm{H}}^{\star }$ Tafel: ${\mathrm{H}}^{\star }+{\mathrm{H}}^{\star }\to {\mathrm{H}}_{2}$ Heyrovsky:${\mathrm{ H}}^{+}+{\mathrm{H}}^{\star }+{\mathrm{e}}^{-}\to {\mathrm{H}}_{2}$
(Alkaline solution) ${2\mathrm{H}}_{2}\mathrm{O}+2{\mathrm{e}}^{-}\to {\mathrm{H}}_{2}+2{\mathrm{OH}}^{-}$	Volmer: ${\mathrm{H}}_{2}\mathrm{O}+{\mathrm{e}}^{-}\to {\mathrm{H}}^{\star }+\mathrm{ O}{\mathrm{H}}^{-}$ Tafel: ${\mathrm{H}}^{\star }+{\mathrm{H}}^{\star }\to {\mathrm{H}}_{2}$ Heyrovsky: ${\mathrm{H}}_{2}\mathrm{O}+{\mathrm{H}}^{\star }+{\mathrm{e}}^{-}\to {\mathrm{H}}_{2}+{\mathrm{OH}}^{-}$

⋆: represents a catalytic active site

**Fig. 4:**
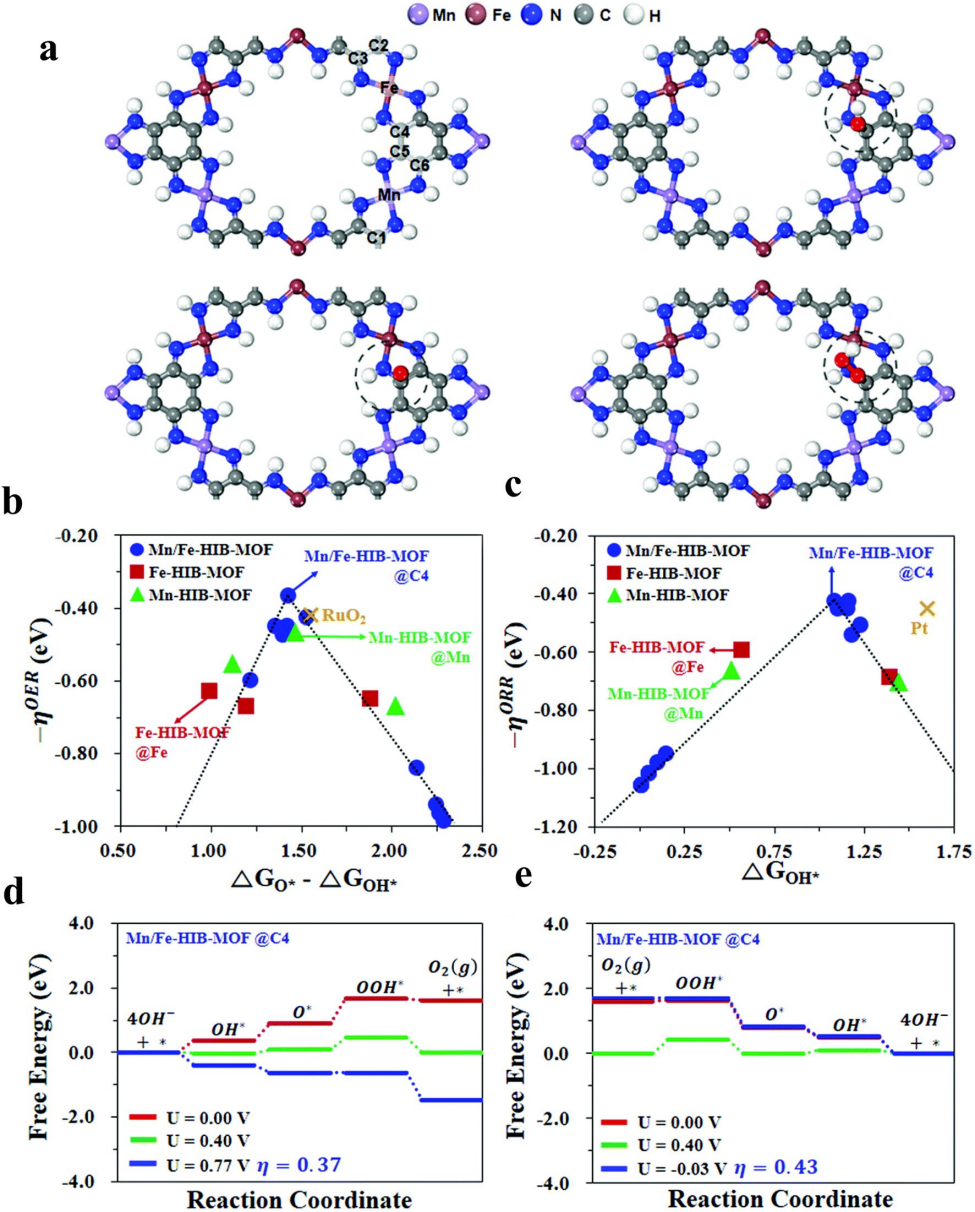
Mechanistic study of bifunctionality for OER and ORR. **a** Initial structure and structures after the adsorption of hydroxyl OH*, oxyl O*, and hydroperoxyl OOH* intermediates on Mn/Fe-HIB-MOF. (Inset: active sites with elements and Arabic numbers.) Volcano profiles for **b** OER and **c** ORR for Mn-HIB-MOF, Fe-HIB-MOF, and Mn/Fe-HIB-MOF catalysts. Free energy diagrams on Mn/Fe-HIB-MOF in an alkaline environment: **d** OER pathways and **e** ORR pathways [106], Copyright © 2019, The Royal Society of Chemistry

One of the central issues in electrochemistry is to design highly active, selective, and stable electrocatalysts at a reduced price through the intimate knowledge of the active sites and reaction mechanism. The principal goal for having a deep understanding is to determine the catalytic activity which can be used to tailor the catalysts atom by atom. Meanwhile, it is particularly challenging to acquire all of the details we need from the experimental techniques. Despite the experimental results that could not give the ideal catalyst expectation, the density functional theory (DFT) exhibits the ability to afford vital mechanistic understandings and predict the promising heterogeneous catalysts. In the electrocatalysis field, DFT is principally revolving around the adsorption energy, binding energy, reaction energy, and reaction barrier. Diverse DFT functionals such as LDA, GGA, meta-GGAs, B3LYP, and other hybrid functions are commonly implemented for calculating different systems since DFT calculations are functional dependent [102, 103]. Nevertheless, through the implementation of DFT calculations in electrocatalysis, there are still three difficulties: (1) the precision of the calculations, (2) the efficacy of the computations, and (3) the intricacy of the environment. The dependability of the DFT results can be determined through the computational accuracy; those numerous advanced methods were settled to have the DFT outcomes more precise; and nevertheless, several features are inadequate owing to the DFT limitations [104, 105]. Conquering these difficulties will let DFT offer more rational designed electrocatalysts. Recently, Shinde et al. [106] did a theoretical study to afford additional perceptions into the root of the Mn/Fe-HIB-MOF intrinsic bifunctional catalytic performance by studying the M-HIB-MOFs OER/ORR electrocatalytic performances and electronic structures (Fig. 4). Theoretically, the overpotential is caused by the potential-determining step, which has the greatest endothermic free energy change in consecutive OER and ORR elementary reaction steps under the standard reaction potential which for OER/ORR in alkaline media is *U* = 0.402 V, as shown in Fig. 4d, e for the green free energy diagrams. Hence, the overpotential donates further potential to the standard reaction potential to increase the catalytic reaction with all energetically advantageous downhill reactions, as exhibited in the diagrams for the blue free energy. The determined M-HIB-MOFs overpotentials versus descriptors exposed that the smallest overpotentials with 0.37 and 0.43 V are for Mn/Fe-HIB-MOF, which surpass those of RuO_2_ + Pt/C (0.42 and 0.45 V), Mn-HIB-MOF (0.53 and 0.64 V), and Fe-HIB-MOF (0.63 and 0.59 V) for OER and ORR, respectively (Fig. 4b, c). It can be concluded that the square planar dual-linked M(II) hexaiminobenzene-MOFs show the ability to be an efficient bifunctional catalyst relating to the overpotential. Consequently, the Mn(II) and Fe(II) dual-linked HIB-MOFs reveal remarkable activity as bifunctional catalytic in an alkaline medium in comparison with Mn-HIB-MOF and Fe-HIB-MOF with electronic characteristics owing to the abundant carbon active sites and improved participation of carbons to the frontier bands. In conclusion, the remarkable efficiency of the bifunctional catalyst and the long-life durability of Mn/Fe-HIB-MOF have been successfully expected and affirmed by the computational analysis.

## Energy Applications of MOF-Derived Electrocatalysts

The fundamental purpose of a well-designed electrocatalyst is to enhance catalytic efficiency as far as feasibility for particular implementations. Due to the functional fragments, morphologies with fine shapes, and advantageous structures, numerous constituents derived from MOFs have been demonstrated as electrocatalysts in the direction of diverse energy conversion methods, in particular the ORR, OER, and HER [102, 107–116]. Remarkably, the MOF-derived materials with unique structures display valuable bifunctional catalytic performance which starts up a novel path for the wide implementation for energy applications, for instance, rechargeable metal–air batteries and water electrolysis. Despite numerous reports that have offered MOF-derived materials for electrocatalysis, a little extraordinary development was done very lately; consequently, an updated progression survey in this area could deliver additional extensive knowledge for the design strategies, DFT calculations, and synthesis protocols for better MOF-derived electrocatalysts.

### MOF-Derived Materials for ORR

#### MOF-Derived Nonmetal Heteroatom-Doped Porous Carbon Electrocatalysts

MOFs crystals contain a large number of carbon-based organic linkers that boost in easing the fabrication of nanocarbon materials. Recently, researchers are dedicating more efforts to developing ORR catalysis based on nonmetal-doped carbon-based materials. Up to now, metal-free electrocatalysts are considered as an exceptional material class with highly functionalized porous heteroatom-doped nanocarbon, categorized as a promising applicant for fuel cells resulting from the exceptional chemical/physical features. Also, they exhibit highly electrocatalytic efficiency, low price, and exceptional stability [88, 117–119]. From the wide range of them, nanocarbon with single-doping (e.g., N–C) [92], nanocarbon with multi-doping (e.g., NS–C, NPS–C) [93, 94], along with nanocarbon hybrids (nanocarbon/CNTs, nanocarbon/graphene) [120], were investigated as exceptional fuel cells electrocatalysts.

##### N-Doped Carbon Electrocatalysts

Obviously, the existence of N-atoms inside the lattice of carbon is able to extensively raise the surface polarity and movement of electron donor inside the frameworks of carbon, producing developed ORR electrocatalytic performance [117, 118, 121, 122]. Nitrogen-doped carbon could be noticed to be the extremely generally explored nanomaterials for the ORR in contrast with heteroatoms. Thanks to the electronegativity variance between C as *X* = 2.55 and N as *X* = 3.06 that polarizes the frameworks of carbon effectively and eases the oxygen adsorption. Furthermore, nitrogen-rich sources are more eco-friendly relative to other heteroatoms. Besides, nitrogen doping modifies the charge distribution and as a consequence enhances the ORR performance. At the catalyst/electrolyte interface, the catalyst–O_2_ adsorption could change from Pauling model (end-on adsorption) to Yeager model (side-on adsorption), resulting in weakening O–O bond, which leads to excess conducive to the approach of the ORR [92].

**Fig. 5:**
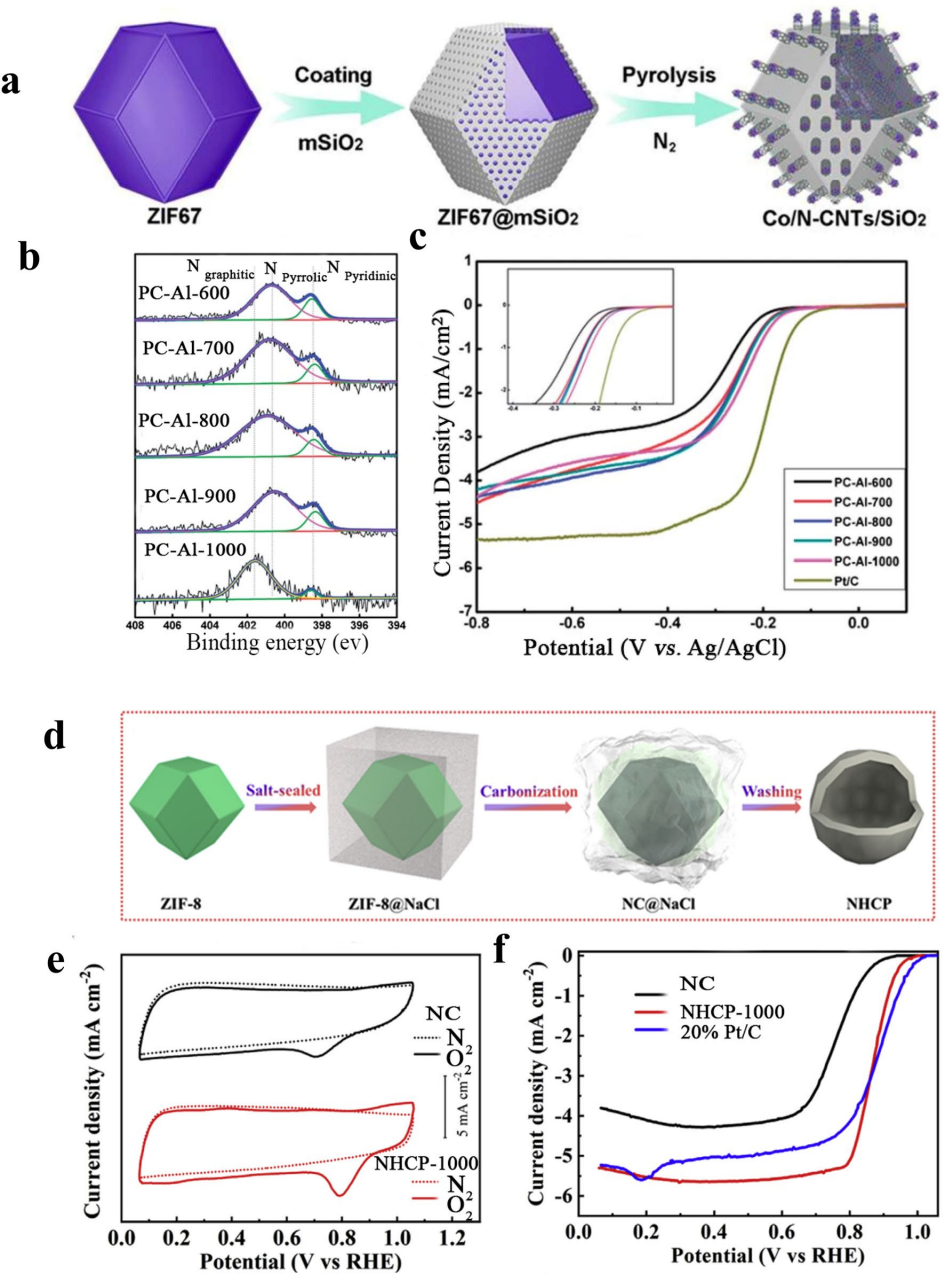
**a** Synthetic procedure of the Co/N-CNTs by the mSiO_2_-coated calcination strategy [19], Copyright© 2018, The Royal Society of Chemistry. **b** XPS spectra of the N 1 s narrow scan of PC-Al-n samples. **c** LSV curves of the carbon products and Pt/C in oxygen-saturated 0.1 M KOH solution at a rotation speed of 1600 rpm and a scanning rate of 10 mV s^−1^ [123], Copyright© 2014, The Royal Society of Chemistry. **d** Schematic illustration of the synthesis of MOF-templated NPS-C-MOF-5 as a metal-free electrocatalyst for the ORR, **e** CVs and **f** LSVs of different samples in O_2_ saturated 0.1 M KOH solution [128], Copyright © 2021, Elsevier

Recently, Yao and coworkers [123] reported carbon-doped nitrogen microporous material synthesis through direct pyrolysis of an amine-functionalized aluminum–MOF compound with amino-MIL-53(Al). Afterward, the obtained sample was dipped in hydrofluoric acid (20%, 5 mL) with stirring for 20 hours for aluminum particles elimination. The correlation between the mesoscopic structures and the electrochemical performance was investigated by studying the carbonization temperatures (*n* = 600, 700, 800, 900, and 1000 °C) for 5 h. XPS was employed to detect the content of nitrogen and the state of PC-Al-n specimens. As shown in Fig. 5b, the XPS shows the details about the conversion of nitrogen from the NH_2_–H_2_BDC amino groups to the pyridinic-N and pyrrolic-N forms at lower temperatures and then the graphitic N state at an elevated temperature. The optimized PC-Al-1000 nanoparticles carbonized at 1000 °C had exceptional ORR electrocatalytic activity at a current density of 0.1 mA cm^2^. Besides, it showed the uppermost onset potential at 0.13 V which is almost like Pt/C (0.07 V) as indicated in Fig. 5c. Obviously, MOFs can be a supreme source to produce N-doped metal-free carbon nanoparticles ORR electrocatalysts.

##### Multiple (Binary and Ternary)-Doped Nanocarbon Electrocatalysts

Lately, numerous studies revealed that multiple doping like phosphorus, boron, sulfur, and nitrogen into carbon can possess exceptional ORR performance [93, 94]. Sulfur atoms are positively charged which is thought to be efficient ORR catalytic sites. Besides, doping with phosphorous can improve the carbon atoms charge delocalization and produce carbon networks including raised edge sites. Current studies have proved that co-doping with heteroatoms for graphitic carbon could positively enhance the ORR performance, which leads to a synergistic effect caused by the charge and spin density differences. This is desirable for oxygen adsorption, electron transfer along with improving the ORR activity [88]. The obtained synergistic impact shows that carbon materials with multi doping might accomplish higher action contrasted with their single-molecule-doped carbon materials [124].

At the same time, ORR electrocatalysts with multi-dopants nanocarbon in particular N-, B-, P-, and S-co-doped carbon can be acquired via changing the organic linkers of MOFs constituent or by rational post-processing processes [125–127]. Dai et al. [127] have reported nitrogen, phosphorous, and sulfur as triple-doped metal-free porous carbon materials. As a template, MOF-5 was used; dicyandiamide (DCDA), dimethyl sulfoxide (DMSO), and triaryl phosphine (TPP) were used as N, S, and P precursors, respectively. In a systematic approach, the influence of the pyrolysis temperature on ORR performance was explored. MOF-5 was pyrolyzed at 700, 900, and 1000 °C. By the way of comparison, the free doping, nitrogen doping, NS, or NP co-doped porous carbon was also employed under the same conditions. In particular, XPS spectra showed that the processing at 900 ℃ of MOF-5/organics leads to an integration of N (4.1%), P (0.27%), and S (0.75%) in the carbon lattice. Likewise, CVs and LSVs have been accomplished to explore the best ORR performances. The optimum temperature appeared to be 900 °C nominated as C-MOF-5-900 which displayed the best ORR onset potential among the studied materials, demonstrating that C-MOF-5-900 had an outstanding electrocatalytic performance. Furthermore, NPS-C-MOF-5 cathodic current density was considerably greater than the signal of the Pt/C electrocatalyst. Particularly, for NPS-C-MOF-5, the peak potential was more positive compared with the other carbon materials, whereas it was insignificantly negative regarding Pt/C electrocatalyst (− 0.13 V). Consequently, these outcomes indicate that NPS-C-MOF-5 was an extraordinary ORR electrocatalyst. But still, more understanding is needed to probe the vital function of the doped carbon on ORR activity which is considerable in designing and optimizing the metal-free doped carbon electrocatalysis. Additionally, Yan et al. [128] obtained nitrogen-doped hollow carbon polyhedrons (NHCP) by directly pyrolyzing ZIF-8 and followed by immersing in NaCl (Fig. 5d). The CVs in Fig. 5e display that both NHCP 1000 and NC catalysts have a clear oxygen reduction peak in O_2_-saturated 0.1 M KOH, compared to N_2_-saturated 0.1 M KOH. Additionally, the cathodic peak of NHCP-1000 (0.79 V) is better than that of NC (0.70 V), signifying a remarkable catalyzing capability for the ORR process of NHCP-1000. Consequently, as indicated in Fig. 5f, NHCP-1000 exhibits outstanding catalytic efficiency with an increased potential (*E*_onset_ = 0.98 V) and half-wave potential (*E*_1/2_ = 0.86 V), analogous to 20% Pt/C (*E*_onset_ = 1.03 V, *E*_1/2_ = 0.88 V).

##### MOF-Derived Nanocarbon Composite Electrocatalysts

**Fig. 6:**
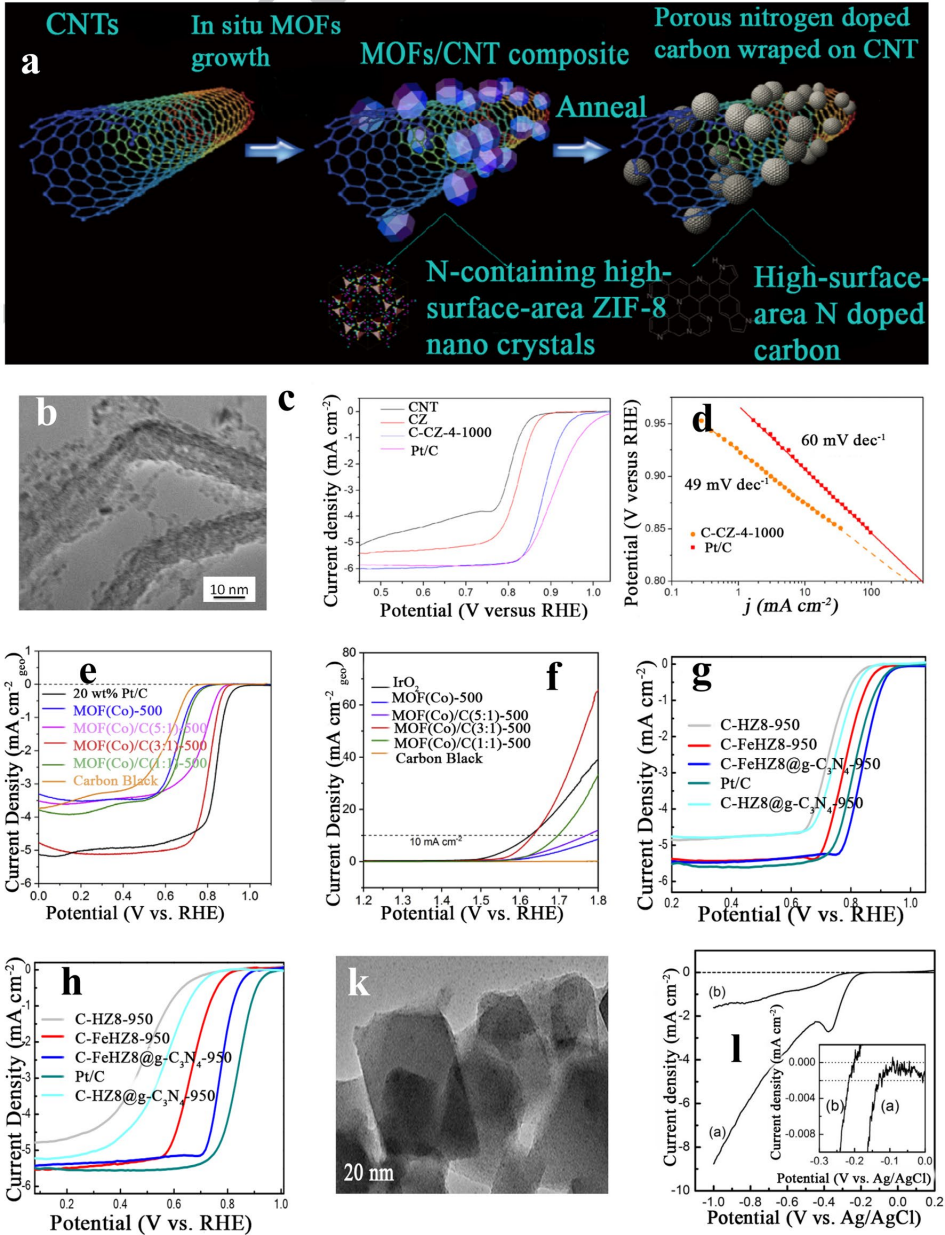
**a** Schematic representations for developing high-surface-area N-doped carbon from MOF/CNT composites, **b** low-magnification TEM image of C-CZ-4-1000, CNT, C-ZIF-1000, **c** C-CZ-4-1000 (0.72 mg catalyst cm^2^) and 40 wt% Pt/C electrocatalysts (80 µg_Pt _cm^2^) at 1600 rpm in O_2_-saturated 0.1 M KOH at 5 mV s^−1^, and **d** Tafel plots of C-CZ-4-1000 and Pt/C [120], Copyright© 2021, Elsevier. **e** ORR LSV curves in the O2-saturated 0.1 M KOH at 1600 rpm with a scan rate of 5 mV s^−1^ and **f** OER LSV curves at 1600 rpm at a scan rate of 5 mV s^−1^ in O2-saturated 0.1 M KOH [135], Copyright© 2019, Elsevier. **g** LSV curves of C-HZ8-950, C-HZ8@g-C3N4-950, C-FeHZ8-950, C-FeHZ8@g-C3N4-950, and 20 wt% Pt/C in **h** O2-saturated 0.1 M KOH and 0.1 M HClO4 (rotation rate: 1600 rpm) [79], Copyright© 2019, The Royal Society of Chemistry. **k** TEM images of the MOF(Fe/Co) sample and **l** ORR activities of the samples: (**a**) MOF(Fe/Co) + SP and (**b**) SP [143], Copyright © 2014, Elsevier

Earlier studies widely explained that the presence of graphene oxide sheets during MOFs formation could successfully form nanocarbon between the graphene oxide layers with a large specific surface area in addition to the superior electronic conductivity [129]. Also, MOFs can rise on carbon nanotubes, accompanied by pyrolysis that could give high catalytic activity toward ORR as have been reported by Zhu et al. [120]. Figure 6a displays the schematic synthesis where ZIF-8 nanocrystals were formed hydrothermally and ORR catalysts based on MOF/CNT composites were employed. MOF/CNT exposed outstanding tolerance to methanol and excellent MOF/CNT electrocatalyst shows a half-wave potential of 24 mV, lesser than Pt/C electrocatalysts. Furthermore, it exhibited a low Tafel slope of 49 mV dec^−1^ in comparison with other electrocatalysts. Owing to the high surface area and nitrogen-functionalized carbon and the exceptional affinity among the thin N-carbon layer and the CNT skeleton, the high ORR electrocatalytic activity was obtained. This composite eases considerably the charge movement, electrical conductivity, and steadiness on the ORR in addition to gas diffusion over the hierarchical porous framework.

#### Monometallic-MOF Derivative-Nanocarbon Electrocatalysts

Metal/metal oxides merging as dopants in porous carbon can enhance the catalytic performance [130, 131]. Moreover, theoretical studies exposed the incorporating metal dopants could remarkably enhance the electron movement from carbon to O_2_ molecules and decrease the O_2_ free energy of adsorption molecules, therefore producing additional ORR active sites. Nevertheless, one of the limiting factors that reduce the implementation of oxides as catalysts in fuel cells is the intrinsic poor conductivity. To overcome this obstacle, covering metal or metal oxide particles with the highly conductive nanocarbon layer (from MOFs pyrolysis) has been studied as an efficient strategy. Lately, transition metal/metal oxide–nanocarbon composite catalysts derived from MOF were broadly investigated, especially Co/Co-oxide and Fe/Fe-oxide nanocarbon. In addition, the obtained highly porous carbon matrix obtained from MOFs can stabilize the ORR performance for M/MO and exhibit fast electron transfer paths and diffusion channels to enhance ORR efficiency.

##### MOF-Derived Cobalt/Cobalt Derivative-Nanocarbon Electrocatalysts

Up till now, Co/Co_3_O_4_ with supported carbon is among the most widely investigated catalysts for ORR in fuel cells [132–134]. Yin and coworkers [135] reported the fabrication of bifunctional electrocatalysis (ORR/OER) by the thermal treatment of a combination of cobalt-based MOF with carbon black (CB). Different ratios between MOF(Co) and CB (1:1, 3:1, and 5:1, respectively) were utilized, and the mixtures were annealed for 2 h in N_2_ at 500 °C. As exposed in Fig. 6e, f, the obtained LSV curves show the enhancement for onset potentials and *E*_1/2_ for the three specimens with added CB than those of MOF(Co)-500. Furthermore, MOF(Co)/C(3:1)-500 with *E*_1/2_ and onset potential are 0.80 and 0.92 V, respectively, and were the highest between them, which were closely resembled of Pt/C catalyst (0.98 and 0.84 V, respectively). Additionally, MOF(Co)/C (3:1)-500 showed current density at 0.76 V (4.10 mA cm^−2^) and the limiting current density (5.12 mA cm^−2^), similar to those of Pt/C catalyst (4.35 and 5.31 mA cm^−2^, respectively), and significantly more than those of the other samples, signifying the outstanding ORR performance of MOF(Co)/C(3:1)-500 to that of other as-sensitized catalysts. The adjusted Co–CoO–Co_3_O_4_/NC displayed outstanding bifunctional performance, robustness, and showed enhanced performance than Pt/C catalyst (for ORR).

##### MOF-Derived Iron/Iron Derivative-Nanocarbon Electrocatalysts

Other transition-metal MOFs, such as Fe-MOFs, were additionally stated as constitutions in the production of Fe-N_x_-C and N-doped Fe/Fe_3_C@C/RGO electrocatalysts to activate ORR [27, 95, 96, 136]. Deng et al. [137] described a hollow N, Fe-doped carbon nanopolyhedron catalyst, obtained through thermal treatment for hollow ZIF-8 with C_15_H_24_FeO_6_ and g-C_3_N_4_. The higher ORR activities are arranged in the order C-HZ8-950 > C-HZ8@g-C_3_N_4_-950 > C-FeHZ8-950 > CFeHZ8@g-C_3_N_4_-950 in 0.1 M potassium hydroxide and 0.1 M perchloric acid. The supreme ORR efficiency of C-FeHZ8@g-C_3_N_4_-950 in the alkaline and acidic media was additionally approved by the LSV curves as displayed in Fig. 6g, h. With the optimized FeHZ8@g-C3N4-950 electrocatalyst, the half-wave potential was larger than commercial Pt/C (30 mV) in an alkaline electrolyte, and only 60 mV lesser than that of Pt/C in an acidic electrolyte (0.78 vs. 0.84 V), similar to the performance of up-to-date Fe–N–C. Furthermore, C-FeHZ8@g-C_3_N_4_-950 displayed outstanding robustness in both acidic and alkaline electrolytes, confirmed via elongated chronoamperometric measurement with 0.78 V in the former and 0.845 V in the latter of the half-wave potential. The current density of Pt/C reduced by 11%, while C-FeHZ8@g-C_3_N_4_-950 missed 4% of its original ORR efficiency (for 5.55 h) and still reserves 91.6% of its initial ORR efficiency for 24 h. Furthermore, CFeHZ8@g-C_3_N_4_-950 reserved more than 93% of its original current density, while the Pt/C displayed a current loss of 35% for 5.55 h. After completing the test for 24 h, C-FeHZ8@gC_3_N_4_-950 reserved 75% of its original ORR activity. Benefiting from the high density of Fe(II)–N_4_–H_2_O active sites, the large surface area, and the hollow porous structure, the catalyst displayed excellent ORR performance and superior robustness in the acidic and alkaline electrolytes.

##### Other MOF-Derived Monometal-Nanocarbon Electrocatalysts

There are numerous classes of metal-based MOFs as Cu-MOFs, Zr-MOFs, Ni-MOFs, and Cd-MOFs that were broadly explored as template precursors to obtain porous metal-nanocarbon electrocatalysts for fuel cells [138–140]. Kim et al. [139] presented a simple process for the efficient filling of Cu- and Ni-HKUST-1. The obtained MOF@mC samples displayed supreme ORR activities compared with the pristine MOFs. Besides, Cu-MOF@mC also displayed noticeable ORR performance, outstanding methanol tolerance, and long-term stability in comparison with the non-Pt-based catalysts for the ORR. Additionally, Cu-MOF@mC exhibited raised *j*_*K*_ values than Ni-MOF@mC at all potentials, strongly demonstrating the noticeable ORR performance of the Cu center. Through this study, it was inducted that large surface area MOFs with well-organized pore constructions and chemical tunability can be hired as an ORR platform for electrocatalysts to obtain conducting 3D networks.

#### Bimetallic-MOFs-Based Electrocatalysts

Two or more kinds of inorganic centers from metal ions or clusters can be incorporated in bimetallic-MOFs, coordinating with different organic ligands, or linkers containing different metals. To obtain a controlled composition, different metals can play an important role [141, 142]. Moreover, the M_1_ and M_2_ coupling is vital for supporting the electrocatalytic performance. By substituting metal or presenting heterogeneity, it would be feasible to adapt the metal sites. Up to 2, 4, 6, 8, and 10 different metal ions types were combined inside one MOF to prepare heterometallic MOF with retaining the original topology, which could open a new avenue in electrocatalysis [54].

##### *Fe/M (M* = *Co or Cu) Bimetallic-MOFs Electrocatalysts*

MOFs consisting of two different metals ions like Fe and Co were reported by Yin et al. [143], and Fe and Cu were also applied as bifunctional electrocatalysis by Wang et al. [17]. The MOF(Fe/Co) catalyst was obtained hydrothermally and showed a fine crystalline structure containing plenty of micropores with a large specific surface area and significant thermal stability. As can be seen, Fig. 6k demonstrates TEM images of MOF(Fe/Co) with a size range of 50–150 nm. The current density at − 2 mA cm^2^ was then indicated for the corresponding onset potential in the ORR process. Figure 6l indicates poor ORR performance and an onset potential of − 0.22 V for SP, whereas it became − .13 V for MOF(Fe/Co) + SP. Likewise, SP exhibited a current density of 0.07 mA cm^2^ at − 0.3 V; however, the current density of MOF(Fe/Co) became 17 times higher than that of SP at the same potential. The reduction process of Fe(III) to Fe(II) was indicated through ORR LSV at − 0.38 V, which proved that MOF(Fe/Co) possessed outstanding ORR/OER dual-function catalytic performance. Results also evidenced that various transition metals, for instance, Fe and Co, are auspicious to the OER and ORR. Furthermore, other factors such as exceptional surface area and microporous morphology of MOF(Fe/Co) exhibited beneficial influences on oxygen diffusion and catalytic site implementation in reaction procedures. Thanks to these considerations, an exceptional dual-function catalytic performance for both OER and ORR of the MOF(Fe/Co) could be obtained.

##### Ni/M Bimetallic-MOFs Catalysts

**Fig. 7:**
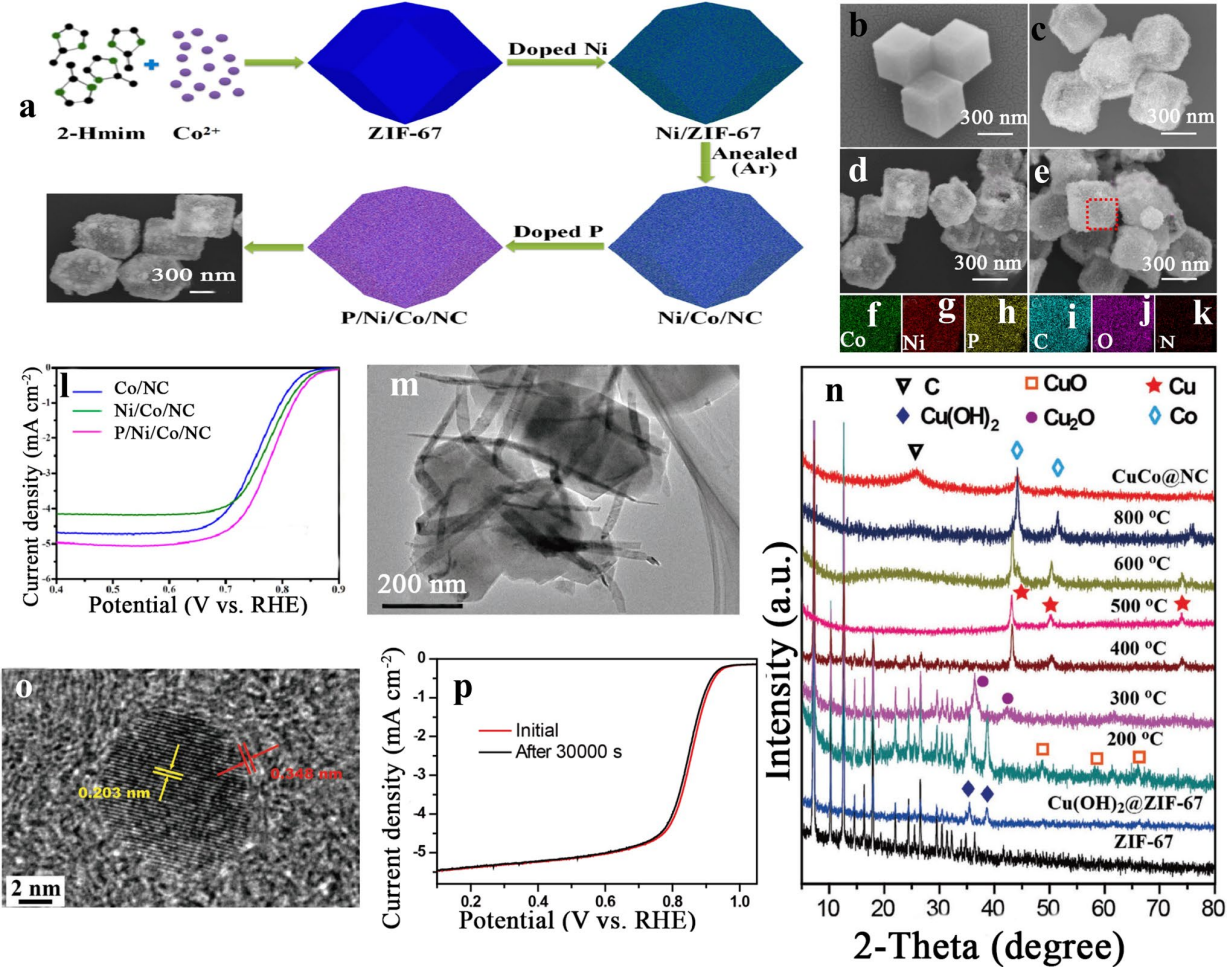
**a** Schematic illustration of the synthesis of Ni/Co/NC electrocatalyst. SEM images of **b** ZIF-67, **c** Ni/ZIF-8, **d** Ni/Co/NC, **e** P/Ni/Co/NC, **f–k** EDS maps of P/Ni/Co/NC and **l** linear sweep voltammetry of Co/NC. Ni/Co/NC, P/Ni/Co/NC in O_2_-saturated 0.1 mol L^−1^ KOH at a scan rate of 10 mV s^−1^ with an RDE rotation rate of 1600 rpm [144], Copyright© 2018, Elsevier. TEM images of ZIF-67 polyhedrons grown on Cu(OH)2 nanowires **m**, XRD patterns of ZIF-67, Cu(OH)2@ ZIF-67, Cu(OH)2@ZIF-67 calcined at different temperatures of 200, 300, 400, 500, 600, and 800 °C, and CuCo@NC **n**, HRTEM image of CuCo@NC composite **o** and LSV curves of CuCo@NC before and after stability test at an RDE rotation rate of 1600 rpm in O2-saturated 0.1 M KOH **p** [145], Copyright © 2017, John Wiley and Sons

Ni/M incorporating in porous carbon was considered a successful method for enhancing the activity for non-noble ORR catalysts. Recently, ZIF-67 was used as a precursor for polyhedral morphology with a porous structure containing Ni/Co, as stated by Chen et al. [144]. Figure 7a shows that P/Ni/Co/NC electrocatalyst was obtained by dispersing ZIF-67 in Ni(NO_3_)_2_·6H_2_O. Afterward, by centrifuged and drying at 60 °C for the mixture, Ni/ZIF- 67 was obtained. To differentiate, annealing under argon gas at 800 °C for 2 h, Ni/Co/NC and Co/NC were obtained from Ni/ZIF-67 and ZIF-67, respectively. Figure 7b shows the scanning electron microscope images of ZIF-67, Co/NC, Ni/Co/NC, and P/Ni/Co/ NC, along with the EDS elemental mappings of P/Ni/Co/NC. Figure 7c shows the SEM image of ZIF-67 particles, exhibiting dodecahedral morphology. Furthermore, Fig. 7d, e indicates the SEM images of Ni/Co/NC. Ni/Co/NC particles preserved the dodecahedral construction of ZIF-67 particles with hollow morphology. Also, when Ni/Co/NC was doped with P element by evaporating technique, it preserved the dodecahedral geometry and the hollow morphology. Figure 7f–k indicates the formation of P/Ni/Co/NC. Also, P/Ni/Co/NC EDS maps were performed for P, Co, Ni, C, O, and N elements. All particles have well-dispersed elements. Figure 7f–k shows the LSV measurements, where the ORR onset potential of Co/NC was 0.847 V corresponding to a current density of 0.1 mA cm^−2^. It was concluded that phosphorous and nickel can enhance the ORR onset potential of Co/NC might be released from the rise of the active sites in the electrocatalyst via doping with phosphorous and nickel elements. Finally, it is feasible for P/Ni/Co/NC to be utilized in actual fuel cells owing to its outstanding ORR activity.

##### Cu/Co Bimetallic-MOFs Electrocatalysis

Theoretically, copper (Cu) is suggested to exhibit high reduction ability close to Pt; nevertheless, the higher preparation temperatures hinder the high diffusion behavior of Cu, which reduces its homogeneous incorporation with carbon. Recently, Zheng et al. [145] overcame this problem by developing a bimetal combination like copper and cobalt implanted in carbon-doped with nitrogen platform (symbolized as CuCo@NC), by means of the in situ growth polyhedrons of ZIF-67 on Cu(OH)_2_ nanowires, followed by thermal treatment of these two precursors. Figure 7p shows the LSV plot of the CuCo@ NC electrocatalyst with an enhanced ORR efficiency. CuCo@ NC gave an increased onset potential of 0.96 V and outstanding *E*_1/2_ of 0.884 V, relative to 1.038 and 0.842 V of 30% Pt/C catalysts, respectively.

### MOF-Derived Materials for OER

Hitherto, OER electrocatalysis is controlled by the presence of transitional metals oxides and hydroxides. Consequently, MOFs open a promising approach through changing into metal oxides efficiently. Lately, OER MOF-derived materials have gained great attention from various research groups [146–148]. This attention thanks to numerous factors, such as (i) there are increased probabilities to obtain new OER active sites through metal–ligand coordination; (ii) different cations replacement in MOFs boosts catalyst activity; and (iii) MOF-derived materials support the design for forming composites with a high surface area. In the present section, we will essentially concentrate on diverse methods that boost the OER performance for the MOF-derived catalysts.

#### Metal-Free OER Electrocatalyst

**Fig. 8:**
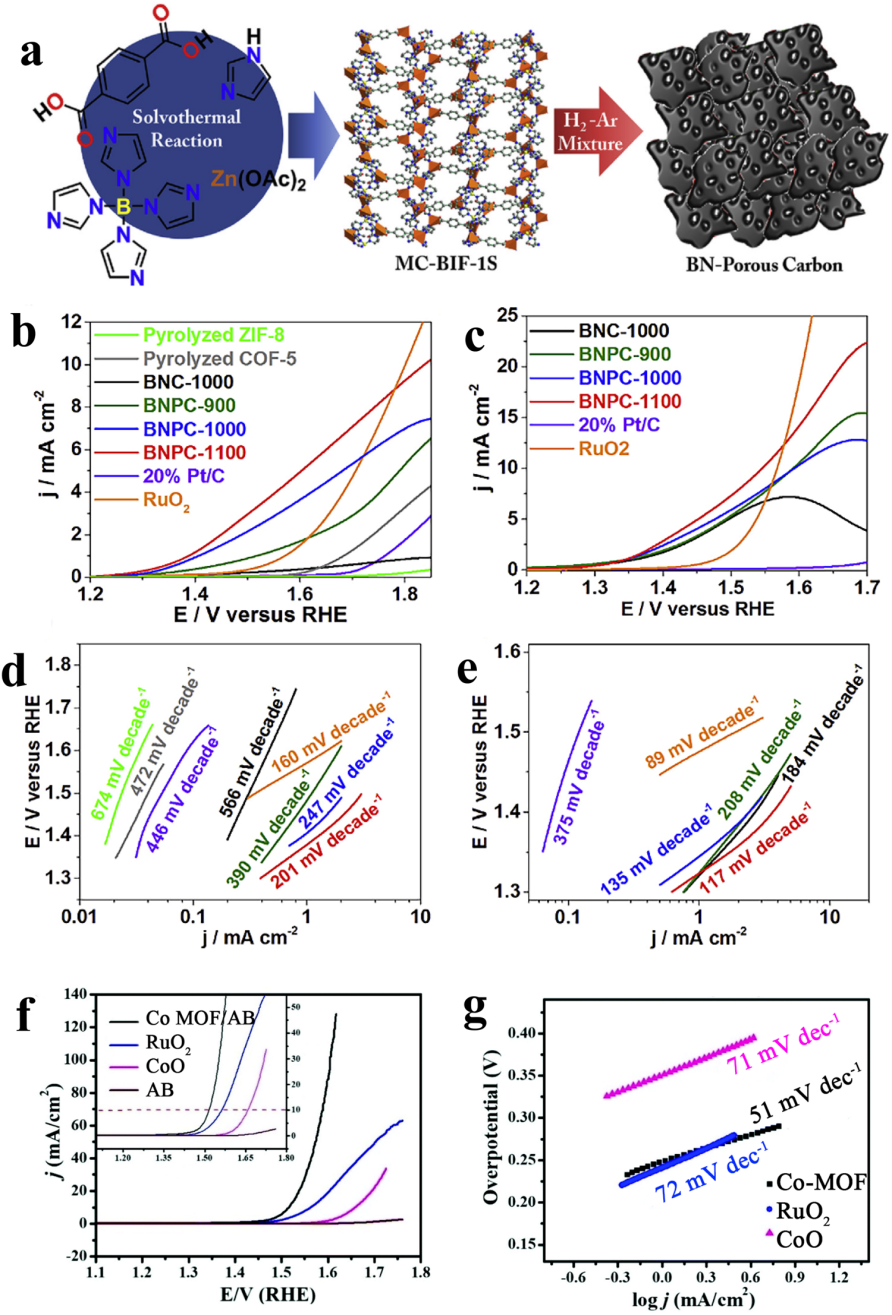
**a** Synthetic scheme of BNPCs, LSV. **b, d** Tafel plots of pyrolyzed non-B-doped MOFs, BNC, BNPCs, 20% Pt/C, and RuO_2_ in 0.1 M KOH and **c, e** 6 M KOH [77], Copyright© 2017, Elsevier. **f** LSVs for the OER by Co-MOF/AB, commercial RuO_2_, CoO (after TGA), and AB in a 1 M KOH electrolyte at the sweep rate of 5 mV s^−1^ and g the corresponding Tafel plot [153], Copyright © 2019, The Royal Society of Chemistry

Transition metals that emerged in porous materials were comprehensively investigated as OER catalysts; nevertheless, they are still restricted by metal particle accumulation and leaking after long-term cycles. Relative to loaded electrocatalysts, electrocatalysts of metal-free carbon do not expose the challenges of particle agglomeration and leaching and consequently can display improved stability throughout long-term usage. Furthermore, it was confirmed that carbon materials doped with one or numerous types of heteroatoms (e.g., nitrogen, phosphorus, sulfur, and boron) are advantageous for their catalytic activity. Qian et al. [77] by pyrolyzing a MOF precursor Zn-MOF (MC-BIF-1S) under H_2_-containing gas, prepared extremely porous boron–nitrogen dual-doped carbon materials. The existence of N and B in the carbon materials (BNPC) and the high porosity were able to efficiently enhance the OER catalytic activity (Fig. 8). As indicated, the uppermost OER electrocatalytic current across all the carbon-based electrocatalysts in that study and BNPC-1100 had the second-lowest slope in the Tafel plots. Thanks to the porosity (859 m^2^ g^−1^) and boron–nitrogen dual-doped chemical composition, BNPC-1100 exhibited outstanding OER catalytic performance. It is common knowledge that the adsorption of OH^−^ and H_2_O is vital to begin the OER pathway; furthermore, nitrogen dopants with positively charged carbon atoms around it along with the boron dopants deliver adequate centers to enhance the electron transfer among electrocatalyst and reactants.

#### MOF-Derived Metal-Involved Electrocatalysts

The hierarchy surface morphology, larger specific surface area, tunable chemical structure, and the existence of the maximum number of active centers make MOFs an appropriate substitute for noble metal-based OER electrocatalysts [131, 149, 150]. This approach can introduce a category of materials that have diverse dimensionalities constructed from organic ligands and metal cations through optimized reaction conditions. Within the majority of the cases, the MOFs are indicated to serve as electrical insulators, which limits their electrochemical implementation. Nevertheless, through optimizing the reaction conditions and the chemical composition, MOFs with high conductivity, and electrocatalytic performances have been investigated by numerous groups. [151, 152] Recently, Tripathy et al. [153] synthesized Co-MOF and studied its catalytic implementation regarding the OER reaction. As shown in Fig. 8f, g, the Co-MOF is performing fine to the OER with inferior onset potential and a small Tafel slope relative to the RuO_2_ electrocatalyst. Furthermore, it required 280 mV overpotential to provide a current density of 10 mA cm^−2^, with strong stabilization. Therefore, it is generally believed that the as-prepared Co-MOF shows the ability to be applied as a cathode and an anode catalyst in miscellaneous future energy applications.

#### MOF-Derived Metal Oxide Catalysts

**Fig. 9:**
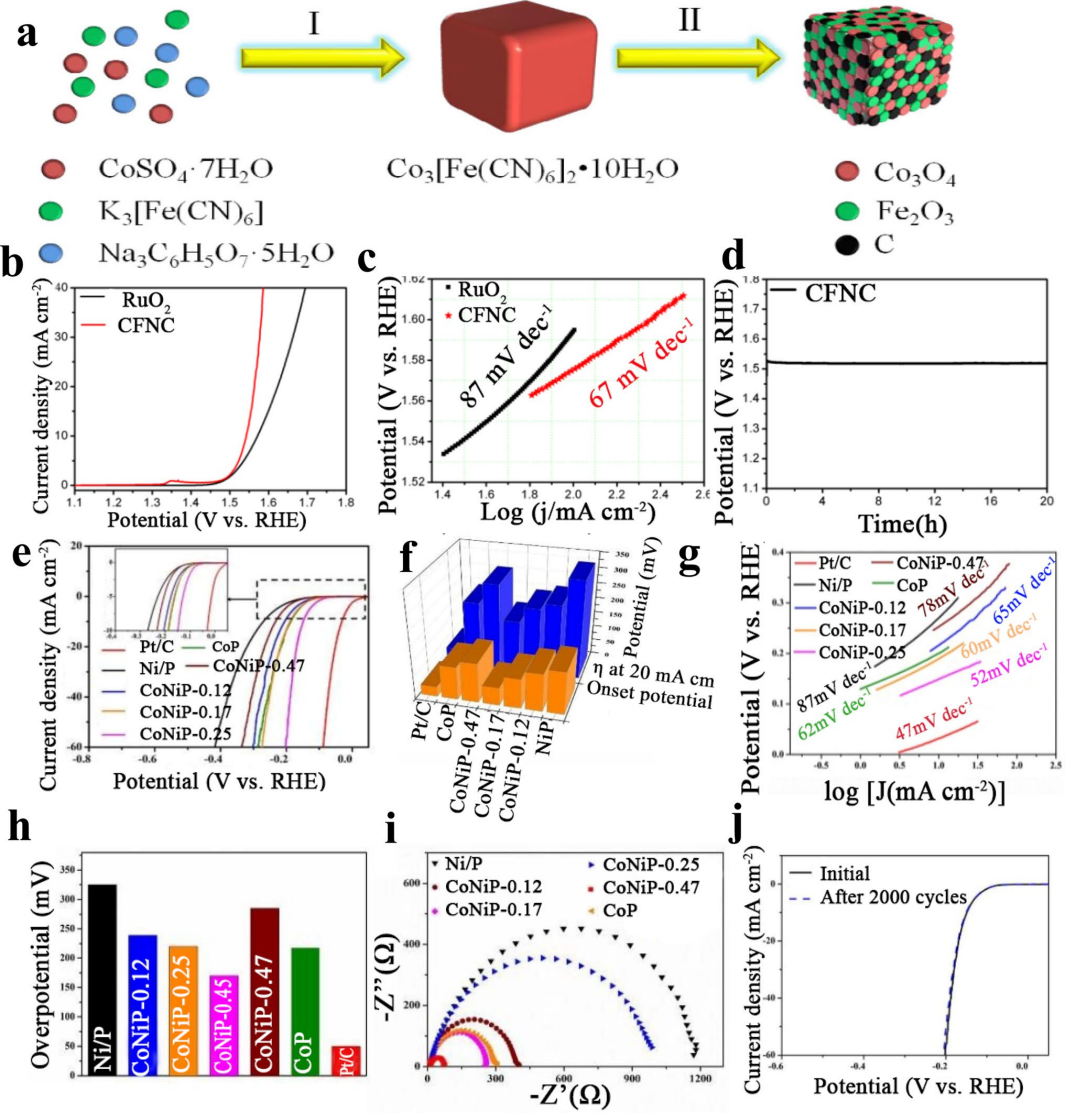
**a** Schematic illustration of the fabrication processes of CFNC: (I) formation of Co_3_[Fe(CN)_6_]_2_·10H_2_O nanocubes; (II) calcinated in an air atmosphere to prepare CFNC; **b** polarization curves of CFNC and RuO_2_, respectively, **c** Tafel plots of CFNC and RuO_2_, respectively, and **d** stability tests of CFNC with 20 h in 1.0 M KOH solution [78], Copyright© 2019, Elsevier. Electrochemical performances of Pt/C, CoP, NiP and CoNiP-n (*n* =  0.12, 0.17, 0.25, 0.47) microspheres: **e** HER polarization curves, **f** histogram of the onset potential and overpotential of the as-prepared electrodes at the current density of 20 mA cm^−2^, **g** the Tafel plots, **h** histogram of the Tafel slopes, **i** Nyquist plots and **j** LSV curves of the CoNiP-0.25 microspheres [164], Copyright © 2019, The Royal Society of Chemistry

As OER catalysts, few metal oxides that emerged in porous carbon derived from MOFs were investigated; nevertheless, more efforts are still needed to discover this area of non-precious metal oxides [154, 155]. Herein, Wei et al. [78] designed a MOF-based (Co_3_[Fe(CN)_6_]_2_·10H_2_O) approach with the aim of obtaining Co–Fe-mixed metal oxide (Co_3_O_4_/Fe_2_O_3_) nanocubes (CFNC) as revealed in Fig. 9a. In the meantime, when CFNC was applied as an electrocatalyst for OER, it needed a low overpotential of 310 mV to exhibit a current density of 10 mA cm^−2^ compared with RuO_2_ (330 mV). Moreover, the CFNC showed extraordinary long-term durability after 20-h duration test (Fig. 9b–e). Also, CFNC and RuO_2_ Tafel plots indicate that the Tafel slope of CFNC (67 mV dec^−1^) was lower than RuO_2_ (87 mV dec^−1^). In addition, Zhou et al. developed a feasible plan that made 2D MOFs act as templates to construct metal oxide/carbon (MO*x*/C, M=Co, Ni, and Cu) nanosheet arrays for OER. Owing to the improved conductivity and additional exposed active sites offered by the 2D structure with the plenty hierarchical pores and the integration with porous carbon, such 2D MOF-derived MO*x*/C arrays showed improved electrocatalytic efficiencies and decent robustness. Particularly, Co_3_O_4_/CBDC, NiO/CBDC, and Cu_2_O/S-CTDC revealed small overpotentials of 208, 285, and 313 mV at the current density of 10 mA cm^−2^, respectively [155]. These catalysts represent a significant complement to the family of MOF-based functional materials besides emphasizing the potential implementation of composite materials in energy conversion and storage systems.

### MOF-Derived Materials for HER

#### MOF-Derived Transition Metal/Heteroatoms (P, S, O, C, Se)

Presently, the most applicable catalyst for HER is platinum (Pt), exhibiting a nearly zero onset overpotential. Nevertheless, the high cost and rare presence in nature restrict its extensive application in industries. Therefore, numerous research groups are promoting much effort in searching for HER electrocatalyst alternatives with exceptional electrochemical performance, better stability, and reasonable prices. Heretofore, the highest first category for the outstanding activity and cheap HER catalysts mainly includes transition-metal phosphides, sulfides, carbides, selenides, and oxides such as MoP [156, 157], NiP [158], NiCoP [159], CoP/MOF [160], Cu_3_P [97], NiFeMoS [161], NiFeCo [162], and MoC [163]. Du et al. [164] designed a new protocol to prepare a sequence of CoNiP hollow microspheres of multi-shelled with various proportions of cobalt to nickel via a metal–organic platform as both the precursor and the template and the as-synthesized CoNiP-0.25 donates a pre-eminent electrocatalytic efficiency for the HER in 1.0 M KOH. As shown in Fig. 9e–j, the CoNiP-0.25 spheres exhibited 20 mA cm^2^ at a potential of 170 mV, which is 120 and 59 mV less than that of pristine NiP and CoP, respectively. The exceptional HER performance of the CoNiP-0.25 spheres can be related to their electronic structure, the desired multi-shelled hollow morphology, and the huge exposure of the active phase bimetallic phosphide CoNiP. Furthermore, the improved HER robustness of CoNiP catalyst could similarly be originated from its unique structure of multi-shelled hollow microsphere, which exhibited its superficial oxidation throughout the catalytic operation.

#### MOF-Derived Metal-Based Electrocatalysts

**Fig. 10:**
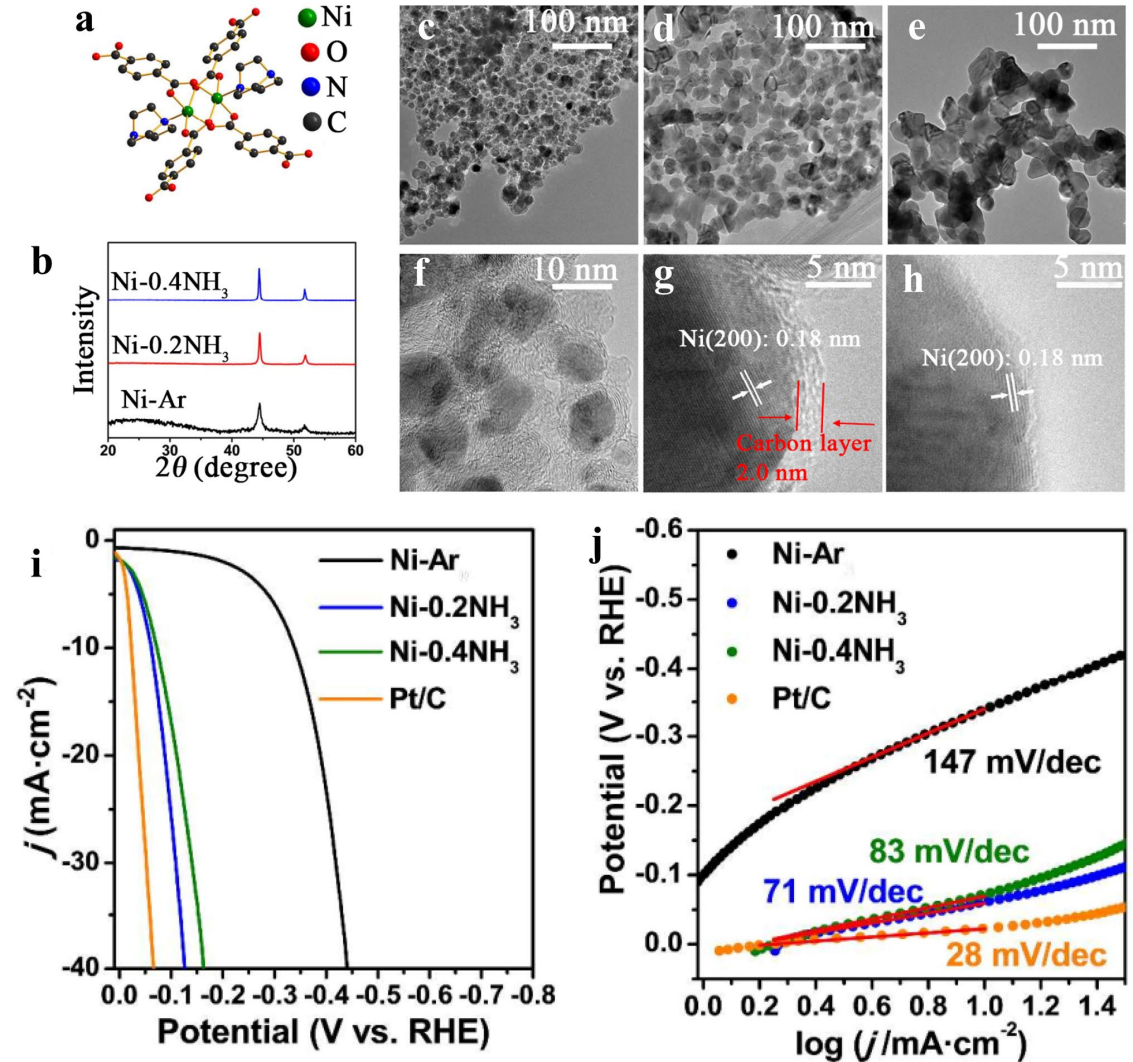
**a** Structure of the Ni coordination environment in Ni_2_(bdc)_2_(ted), **b** XRD patterns of the Ni-MOF-derived catalysts, **c–h** TEM images of the Ni-MOF-derived catalysts, **i** linear scanning voltammograms, and **j** Tafel plots of the MOF-derived catalysts in 1 M KOH solution [166], Copyright © 2015, The Royal Society of Chemistry

**Fig. 11:**
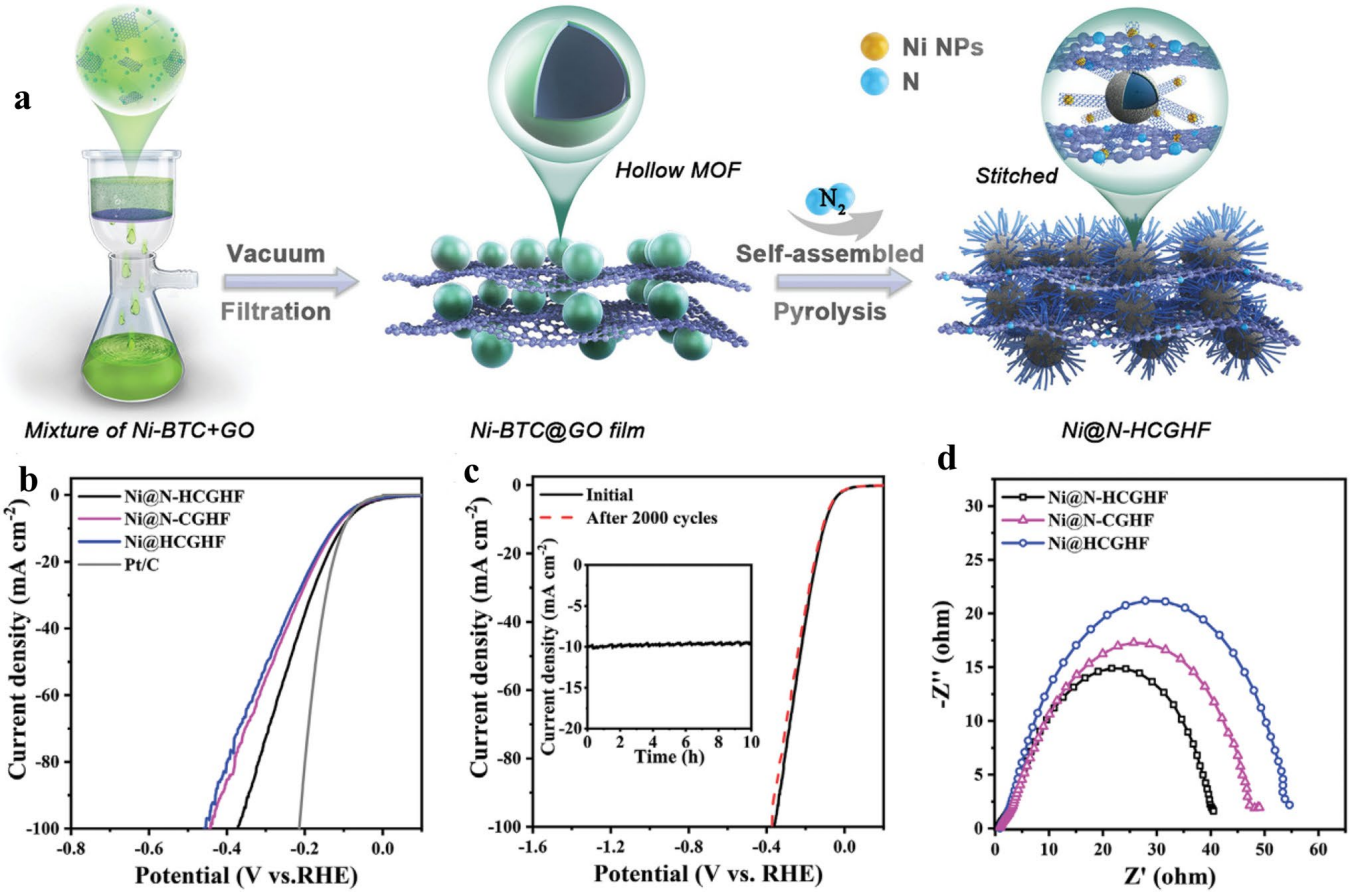
**a** Schematic of the preparation of Ni@N-HCGHF, **b** LSV curves for HER of various samples. **c** LSV curves for HER of Ni@N-HCGHF before and after 2000 CV cycles (inset: the current–time curve of Ni@N-HCGHF). **d** EIS Nyquist plots [30], Copyright © 2020, John Wiley and Sons

Recently, MOFs-incorporated metals attract researchers’ attention [165, 166]. Wang et al. [166] well designed MOF structure through pyrolysis by using ammonia. The Ni-MOF pyrolysis in ammonia reduced nickel nanoparticles with surface nitridation and thin carbon coating films. As shown in Fig. 10, the electrocatalysts have been gotten in NH_3_, indicating an outstanding structural difference. As can be seen, the Ni–0.2NH_3_ specimen included only extremely thin carbon layers of 2 nm covering the surface of the Ni particles (Fig. 10f), whereas the Ni–0.4NH_3_ specimen exhibited almost no carbon covering. Both specimens gained in the NH_3_ atmosphere included Ni particles of about 30–50 nm (Fig. 10e, g). The adapted Ni nanoparticles surface-displayed at a current density of 20 mA cm^2^ low overpotential of only 88 mV (Fig. 10j). The findings propose that tuned thermal treatment of MOFs is an efficient avenue to obtain exceptional well-designed noble metal-free HER catalysts. In this consequence, Yan et al. [30] prepared 3D heterostructure film from a Ni-centered MOF/graphene oxide, as shown in Fig. 11a. As shown in Fig. 11b, the Ni@N-HCGHF displays an outstanding HER activity with a small overpotential of 95 mV (*η*10) at 10 mA cm^−2^. Figure 4c indicates a small decay of the Ni@N-HCGHF, which can be detected after 2000 CV cycles and the *i–t* plot (inset of Fig. 4b) for the HER displays that the Ni@N-HCGHF electrode preserves 94.5% of the original HER activity after 10 h. Additionally, the Ni@N-HCGHF exhibits the smallest charge transfer resistance which proves that the fastest charge transfer kinetics and more active sites on the surface are for Ni@N-HCGHF (Fig. 11d). The findings obtained from the experiments with the theoretical calculations showed that the synergistic effect of the N-doped carbon shell and Ni nanoparticles can lead to an optimized film with excellent electrocatalytic activity, demonstrating the possibility of the film for real implementations.

## Conclusions and Perspectives

Designing for highly active and economic electrocatalysts becomes a vital part of current studies and becomes a core of interest for numerous researchers. Numerous factors can be taken into consideration during designing an ideal catalyst such as high activity, low-cost, broadly available, huge surface area, optimized porosity for mass transport, and having a large number of exposed active sites. The foremost classes of non-noble catalysts contain metal-free carbon-based materials, graphene, carbon nanotubes, macrocyclic materials, metal-decorated soft and hard substrates, and metal oxides. Among several non-noble electrocatalysts, the MOF-based electrocatalysts have been grown dramatically in the last recent years with auspicious catalytic properties for important electrochemical reactions owing to their adaptable design. From MOF precursors, the whole of the aforementioned kinds can be gained. Pure MOFs imitate the macrocyclic structures and present ideal platform materials to originate metal-free carbon, carbon nanotubes, metal/carbon composite, and metal oxide nanostructures. Thus, the next-generation materials could be obtained from MOFs; due to the depending on reaction conditions, the feasibility of adjusted tailor capability of building units (organic ligands and metallic centers) could be controlled.

In this report, a summary of MOFs and MOF-based substances for electrocatalysis implementations is stated (Tables 2 and 3). There is a significant increase in using MOFs for ORR, OER, and HER because of their tunable holes, surface area, and the existence of electroactive metal species. Nevertheless, obtaining efficient electrocatalysis is challenging and basic knowledge about electrocatalysis requirements is needed. One of the main challenges that restrict using the as-prepared MOFs is the poor conductivity, which could be overcome by pyrolysis. Before, during, and after pyrolysis, several techniques have been applied to obtain ideal electrocatalysis efficiency for ORR, OER, and HER (Fig. 3). Before pyrolysis, doping with several metals during the formation of MOFs could show efficient electrocatalysis. Likewise, embedding with metal/metal oxide nanoparticles before pyrolysis is another method to obtain carbon nanocomposites. Meanwhile, during pyrolysis, passing gasses like ammonia can produce carbon nanoparticles doped with N-heteroatoms derived from MOFs. Carbon metals-free derived from pyrolyzed MOFs could be obtained by acid etching to dissolve metallic centers involved in the pyrolyzed MOFs. In brief, in the past decade, substantial progress has been devoted to the MOF composites and derivatives in energy applications. With increasing interest, we anticipate more progress toward the practical energy systems of MOF-based materials.

**Table 2:** Summary of the MOF-derived ORR electrocatalysts reported in recent studies

MOF used	Doped element	Electrolyte	*E*_onset_ (V)	*E*_1/2_ (V)	References
ZIF-8	N	0.1 M KOH	0.92 (vs. RHE)	0.82	[125]
ZIF-7	N	0.1 M KOH	0.86 (vs. RHE)	0.7	[168]
MOF-5	N	0.1 M KOH	− 0.051 (vs. Ag/AgCl)	0.171	[169]
MOF-74	N	0.1 M KOH	1.02 (vs. RHE)	0.902	[170]
ZIF-8	N	0.1 M KOH	0.881 (vs. RHE)	0.822	[171]
ZIF-8	N	0.1 M KOH	0.06 (vs. Ag/AgCl)	0.103	[172]
Amino-MIL-53(Al)	N	0.1 M KOH	− 0.13 (vs. Ag/AgCl)	–	[173]
ZIF-67, ZIF-L	N, Co	0.1 M KOH	–	0.822 (vs. RHE)	[174]
ZIF-8	N, Mo, MoS_2_	0.1 M KOH	0.90 (vs. RHE)	0.81	[18]
MOF-5	N, P, S	0.1 M KOH	− 0.006 (vs. Ag/AgCl)	–	[127]
UIO-66-NH_2_ MOF	N, Fe, S	0.1 M KOH	0.97 (vs. RHE)	0.87	[175]
Co-MOF	N, Co, S	0.1 M KOH	0.843 (vs. RHE)	0.782	[176]
Fe-MOF	N, Fe	0.1 M KOH	0.84 (vs. RHE)	0.68	[95]
Co-MOF	N, P, Co	0.1 M KOH	0.88 (vs. RHE)	0.80	[177]
Co–Fe-MOF	N, Fe, Co	0.1 M KOH	0.907 (vs. RHE)	0.817	[28]
Co–Zn-ZIF	N, Co, Zn	0.1 M HClO_4_	0.88 (vs. RHE)	0.78	[178]
ZIF-67	Cu, Co, N	0.1 M KOH	0.96 (vs. RHE)	0.884	[145]

**Table 3:** Summary of MOF-based electrocatalysts for OER and HER in recent publications

MOF used	Doped element	Electrolyte	*E*_onset_ (mV) versus RHE	Tafel slope (mV Dec^−1^)	*E*_*j*=10_ (mV)^a^ versus RHE	References
*MOF-based electrocatalysts for OER*						
MOF	Fe, Ni	1.0 M KOH	–	58	320	[179]
ZIF	Co, Fe, Ni	1.0 M KOH	–	43.75	216	[180]
Zn-MOF	N, B, P	6 M KOH	138	89	117	[77]
Co-MOF	Co	1.0 M KOH	125	51	280	[153]
MOF-based (Co_3_[Fe(CN)_6_]_2_·10H_2_O)	Co_3_O_4_, Fe_2_O_3_	1.0 M KOH	–	67	310	[78]
*MOF-based electrocatalysts for HER*						
Ni-MOF	Ni	1.0 M KOH	–	71	61	[166]
CoNiP-MOF	Co, Ni, P	1.0 M KOH	96	52	174.4	[164]

^a^: *E*_j = 10_ for overpotential required for the current density of 10 mA cm^−2^

Based on this understanding, in addition to the status of up-to-date studies on MOF-derived electrocatalysis, the following points can be taken into account to design good and promising MOF-derived ORR, OER, and HER catalysts:

1. Via intrinsic MOFs and MOF-derived materials, different MOFs including ZIFs and MILs need to be extensively extended to accomplish the necessitates of modern catalysts structures. Additional protocols and synthesis methods for MOF-based materials should be explored to adapt the constitutions, morphologies, and structures to obtain more optimized materials. Additional properties could be explored for indicating the reaction mechanisms and maximizing the synthesis knowledge to reduce the expenses, beneficial to improve the activity and stability of the materials.
2. Theoretically, computational calculations and structural designing should be implemented to know the requirements of particular reactions and decreasing repetition for trial-and-error endeavors. Up till now, although well-established DFT approaches have been established and applied for numerous structures, the DFT direct endorsement in MOF-derived electrocatalysis is still rare and further investigation is demanded. At the same time, thanks to the multi-function performance of MOF materials, it is expected that such area will remain to enlarge and cross with researches, other than those constrained to up-to-date like ORR, OER, and HER electrochemical reactions.
3. Multi‐doping is an encouraging approach to designing high-efficiency MOF-derived electrocatalysts. More consideration is needed to adapt the dopants configuration. Doping with diverse dopants can produce numerous synergetic effects for ORR, OER, and HER electrocatalysis.
4. Under the ORR/OER/HER reaction conditions, the chemical and thermodynamic stability shall be considered for MOF-derived electrocatalysts. Therefore, a high level of graphitization in the carbon matrix is preferred to maintain the high conductivity and stability of the catalysts.
5. In terms of advanced characterization techniques and theoretical calculations, systematized researches will offer a novel perspective to detect the intrinsic catalytic active sites and numerous new possibilities for the rational design and performance revolutions for ORR/OER/HER electrocatalysts.
6. Though significant progress has been attained recently, MOF‐derived electrocatalysis is still far away from utilization in industrial‐scale energy applications toward fuel cells and water electrolyzes. We believe that the continuous development of nanotechnology and electrochemical science, characterizations, and theoretical calculations will conduct the multi‐scale design and synthesis of MOF‐derived materials with optimal activity and robustness.
